# Causality of the gut microbiome and atherosclerosis-related lipids: a bidirectional Mendelian Randomization study

**DOI:** 10.1186/s12872-024-03804-3

**Published:** 2024-03-02

**Authors:** Da Teng, Wenjuan Jia, Wenlong Wang, Lanlan Liao, Bowen Xu, Lei Gong, Haibin Dong, Lin Zhong, Jun Yang

**Affiliations:** 1 Yantai Yuhuangding Hospital Affiliated to Qingdao University, Yantai, Shandong People’s Republic of China; 2 Qingdao University, Qingdao, Shandong People’s Republic of China; 3 Binzhou Medical University, Yantai, Shandong People’s Republic of China; 4 Dazhou Central Hospital, Dazhou, Sichuan People’s Republic of China

**Keywords:** Lipoproteins and Apolipoproteins, Intestinal flora, Atherosclerosis, Mendelian randomization analysis

## Abstract

**Aims:**

Recent studies have indicated an association between intestinal flora and lipids. However, observational studies cannot indicate causality. In this study, we aimed to investigate the potentially causal relationships between the intestinal flora and blood lipids.

**Methods:**

We performed a bidirectional two-sample Mendelian Randomization (MR) analysis to investigate the causal relationship between intestinal flora and blood lipids. Summary statistics of genome-wide association studies (GWASs) for the 211 intestinal flora and blood lipid traits (*n *= 5) were obtained from public datasets. Five recognized MR methods were applied to assess the causal relationship with lipids, among which, the inverse-variance weighted (IVW) regression was used as the primary MR method. A series of sensitivity analyses were performed to test the robustness of the causal estimates.

**Results:**

The results indicated a potential causal association between 19 intestinal flora and dyslipidemia in humans. Genus *Ruminococcaceae*, *Christensenellaceae, Parasutterella, Terrisporobacter, Parabacteroides,* Class Erysipelotrichia, Family Erysipelotrichaceae, and order Erysipelotrichales were associated with higher dyslipidemia, whereas genus *Oscillospira, Peptococcus, Ruminococcaceae UCG010, Ruminococcaceae UCG011, Dorea,* and Family Desulfovibrionaceae were associated with lower dyslipidemia. After using the Bonferroni method for multiple testing correction, Only Desulfovibrionaceae [Estimate = -0.0418, 95% confidence interval [CI]: 0.9362–0.9826, *P* = 0.0007] exhibited stable and significant negative associations with ApoB levels. The inverse MR analysis did not find a significant causal effect of lipids on the intestinal flora. Additionally, no significant heterogeneity or horizontal pleiotropy for IVs was observed in the analysis.

**Conclusion:**

The study suggested a causal relationship between intestinal flora and dyslipidemia. These findings will provide a meaningful reference to discover dyslipidemia for intervention to address the problems in the clinic.

**Supplementary Information:**

The online version contains supplementary material available at 10.1186/s12872-024-03804-3.

## Introduction

At present, cardiovascular disease (CVD) remains the primary contributor to the marked upswing in global mortality [1]. According to the latest statistical data from NHANES, the current overall prevalence of CVD is 49.2%, with the number of affected individuals reaching staggering 126.9 million [2]. Dyslipidemia is an important driver of CVD progression. Elevated plasma concentrations of LDL cholesterol (LDL-C) and triglycerides (TG) and low concentration of HDL cholesterol (HDL-C) are leading contributors to an increased risk for CVD [3–5]**.** What's more crucial is that lipoproteins, as particles with complex compositions, include apolipoproteins as indispensable and vital components [6]. Apolipoproteins can be widely involved in a variety of pathophysiological processes such as atherosclerosis formation [7–10]. Therefore, lowering plasma lipoprotein levels will undoubtedly reduce the incidence of CVD [11]. At this stage, although statin therapy has achieved remarkable and brilliant success [12], researchers are still searching for new therapeutic approaches to combat CVD, in which the close relationship between intestinal flora and lipid levels has attracted increasing attention.

The intestinal flora consists of approximately 4 × 10^13^ commensal bacteria, also known as the “human second genome” [13]. Interventions on intestinal flora have become an important breakthrough in improving health [14, 15]. Fecal transplantation in rodents suggests that the intestinal flora holds promise for treating chronic diseases [16, 17]. Likewise, the important role of intestinal flora in CVD is becoming apparent. Takuo’s study demonstrates a correlation between coronary heart disease incidences and intestinal flora changes [18]. Xuzhi Wan et al.find that changing the abundance of certain intestinal flora affects blood lipid levels to some extent [19]. *Prevotella* and *Bacteroides* in men and *Akkermansia* and *Escherichia/Shigella* in women may be associated with blood lipid levels in an observational study in Japan [20]. However, most of these studies are observational, and the results may be confounded by reverse causality or confounding factors such as diet and antibiotics, making the conclusions less reliable.

Mendelian Randomization (MR) analysis is an important method to explore the causal relationship between exposure and outcome by using genetic variants as instrumental variables (IVs) [21]. Its obvious advantage is that it can avoid the interference of confounding factors in traditional observational studies [22, 23]. This is particularly fundamental in inferring causality. MR analysis can more reliably infer the causal relationship between intestinal flora and blood lipids. On this basis, we performed a two-sample MR analysis to investigate the causal relationship between intestinal flora and blood lipids. This may provide new treatments such as probiotic therapy, dietary modification, and fecal microbiota transplantation (FMT) for CVD in the future.

## Materials and Methods

### Study design

**Fig. 1:**
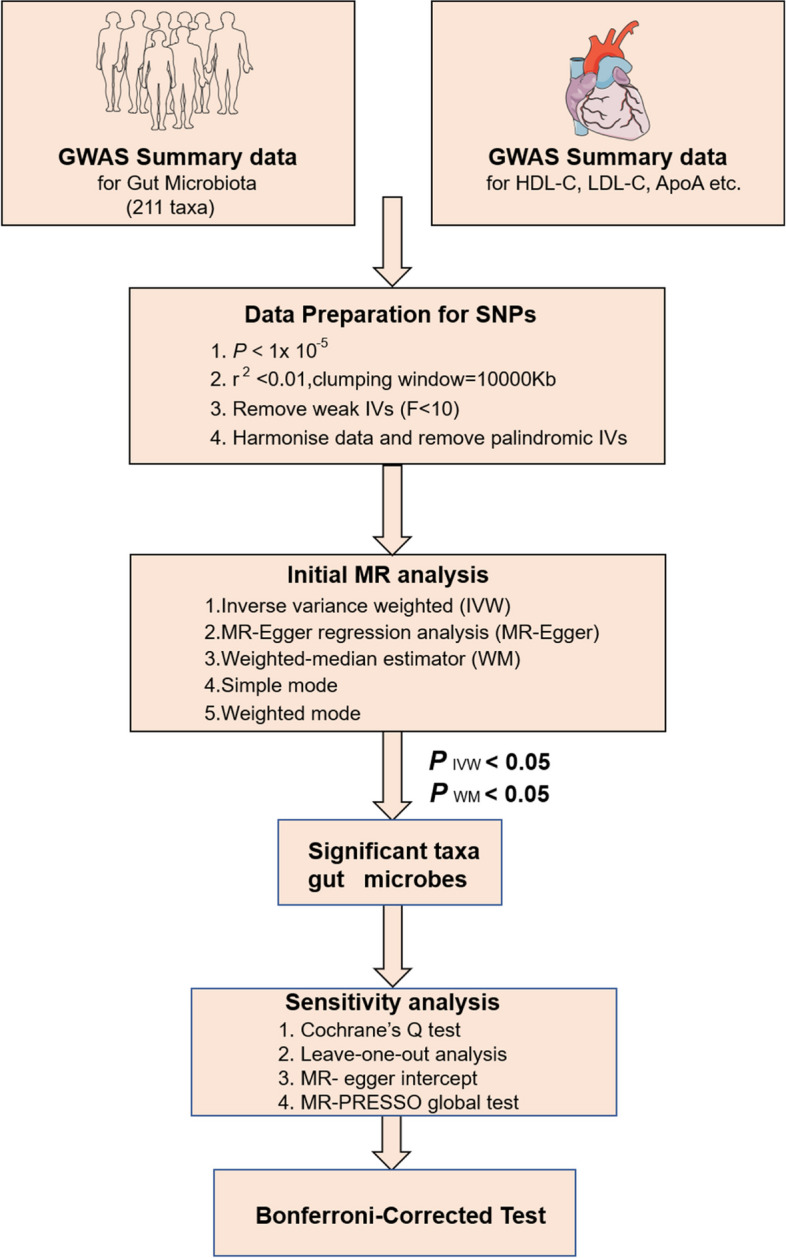
Design of the study. We obtained raw data on intestinal flora and lipids from the GWAS database. The statistically significant intestinal flora was then screened according to P _IVW_< 0.05 and P _WM_< 0.05 and subjected to detailed analysis and reverse MR studies. *F* F-statistics. *r2* the genetic variants for instrument, *IVs* instrumental variables

As shown in Fig. 1, this study was based on a two-sample MR approach to explore the causal relationship between intestinal flora and blood lipids. Compared with single-sample MR, two-sample MR did not need to obtain individual genetic data but used summary statistical information from genome-wide association studies (GWAS) for analysis.

### Data sources

Summary statistics for intestinal flora taxa were obtained from a large-scale multi-ethnic GWAS meta-analysis from 24 cohorts with 18,340 individuals [24]. A total of 211 taxa (131 genera, 35 families, 20 orders, 16 classes, and 9 phyla) were included. For the outcomes – serum lipids—summary statistics were obtained from a GWAS study that included up to 441,016 participants from UKBB [25], available from IEU Open GWAS Database (IEU OpenGWAS project (mercies. ac. UK)).

The GWAS summary data for intestinal flora and serum lipids are obtained from a database of samples collected from European populations, with minimal potential for sample overlap. The source of the data was also approved by the appropriate ethics committee, and therefore no ethical approval was required for this study [25].

### Instrumental variables selection

We performed rigorous quality control (QC) on the single-nucleotide polymorphisms (SNPs) in the microbiota-based GWAS summary studies to select valid IVs for MR analysis. Firstly, the IVs chosen must have a strong association with the exposure, we selected SNPs associated with each genus at the locus-wide significance threshold (*P* < 1.0 × 10^–5^) as potential IVs [26]. Secondly, we clustered SNPs according to the European 1000 Genomes Project reference panel (r^2^ < 0.01, clump distance > 10,000 kb) to identify independent SNPs [27]] Thirdly, we excluded palindromes and incompatible SNPs when harmonizing exposure and outcome statistics. Fourthly, to avoid the effect of weak instrumental bias on causal inference, we also calculated the F-statistic [28]. SNPs with F-values less than 10 were excluded [29].

### MR analysis

We used MR analysis to analyze the causal relationship between intestinal flora and serum lipids. Five prevalent MR methods were employed to estimate the associations between 211 selected intestinal microbiota IVs and each outcome: inverse-variance weighted (IVW) test [30], MR-Egger regression [31], weighted median (WM) [31], Simple mode, and weighted mode [32]. Of these, we used IVW as the primary method of analysis because, without horizontal pleiotropy, the IVW method of analysis would be unbiased [33]. In addition, WM is an important complementary method, which assumes that less than 50% of IVs have horizontal pleiotropy [34].This makes the reliability of the results more robust. If the MR analysis results were nominally significant (*P* < 0.05), we considered that there might be a causal relationship between the intestinal flora and the lipids. The results were considered robust when two or even more MR analysis methods including IVW and WM were significant [35].

We conducted a further sensitivity analysis to make our results more reliable. Conchrane's Q test was used to detect heterogeneity among IVs. Leave-one-out sensitivity analysis was also used to detect potentially influential IVs [36]. Furthermore, to ascertain that the outcomes are influenced by genetic variation rather than other biological pathways, excluding the impact of horizontal pleiotropy, we employed a variety of methods to detect possible horizontal pleiotropy. MR- egger intercept test and global test for outliers (MR-PRESSO) were used to assess the presence of horizontal pleiotropy and results were considered unaffected by horizontal pleiotropy if *P* > 0.05 [37, 38]. The MR-PRESSO outlier test can be used to moderate horizontal pleiotropy by detecting and removing outliers [38].

To obtain a more rigorous interpretation of causality, we performed a Bonferroni correction based on the number of bacteria under each attribute [genera: 0.05/131 (3.8 × 10^−4^), families: 0.05/35 (1.4 × 10^−3^), orders: 0.05/20 (2.5 × 10^−3^), classes: 0.05/16 (3.1 × 10^−3^), and phyla: 0.05/9 (5.5 × 10^−3^)]. Finally, we also performed reverse MR analysis. We treat the significant intestinal flora in two-sample MR studies as the outcome and lipids as the exposure to verify the existence of reverse causality. All statistical analyses were conducted using R (Version 4.1.2) with the two-sample MR [39] and MR-PRESSO packages [38].

## Results

### Instrumental variable selection and initial MR analysis results

The results obtained after the initial analysis of 211 intestinal flora are shown in Supplementary Table S1. We used five MR methods to analyze the causal relationship between intestinal flora and the different lipids in the serum. Using the IVW approach (*P* < 0.05), we initially screened 65 intestinal flora for a potential causal relationship with blood lipids (Supplementary Table S2). Subsequently, among the significant findings, we conducted a double verification using the IVW method integrated with the WM method and observed that only 19 intestinal flora exhibited more robust results in the analysis. (Supplementary Table S3-4). Our study primarily centered on these 19 specific intestinal flora. All SNPs F values exceed 10 (Supplementary Table S5); hence, the existence of a weak instrumental bias is not considered.

### Detailed Two-Sample MR results

#### ApoA

Sixteen intestinal flora were identified as causally related to ApoA through screening with the IVW method (Supplementary Table S2). However, upon integration with the WM method, we inferred that only the genus *Ruminococcaceae* exhibited a suggestive causal relationship with higher ApoA [Estimate = 0.0513, 95% confidence interval [CI]: 1.0238–1.0823, *P* = 0.0003] (Table 1). MR-Egger intercept test and MR-PRESSO global test suggested that there was no horizontal pleiotropy or outliers (*P* > 0.05) (Table 3).

**Table 1.**
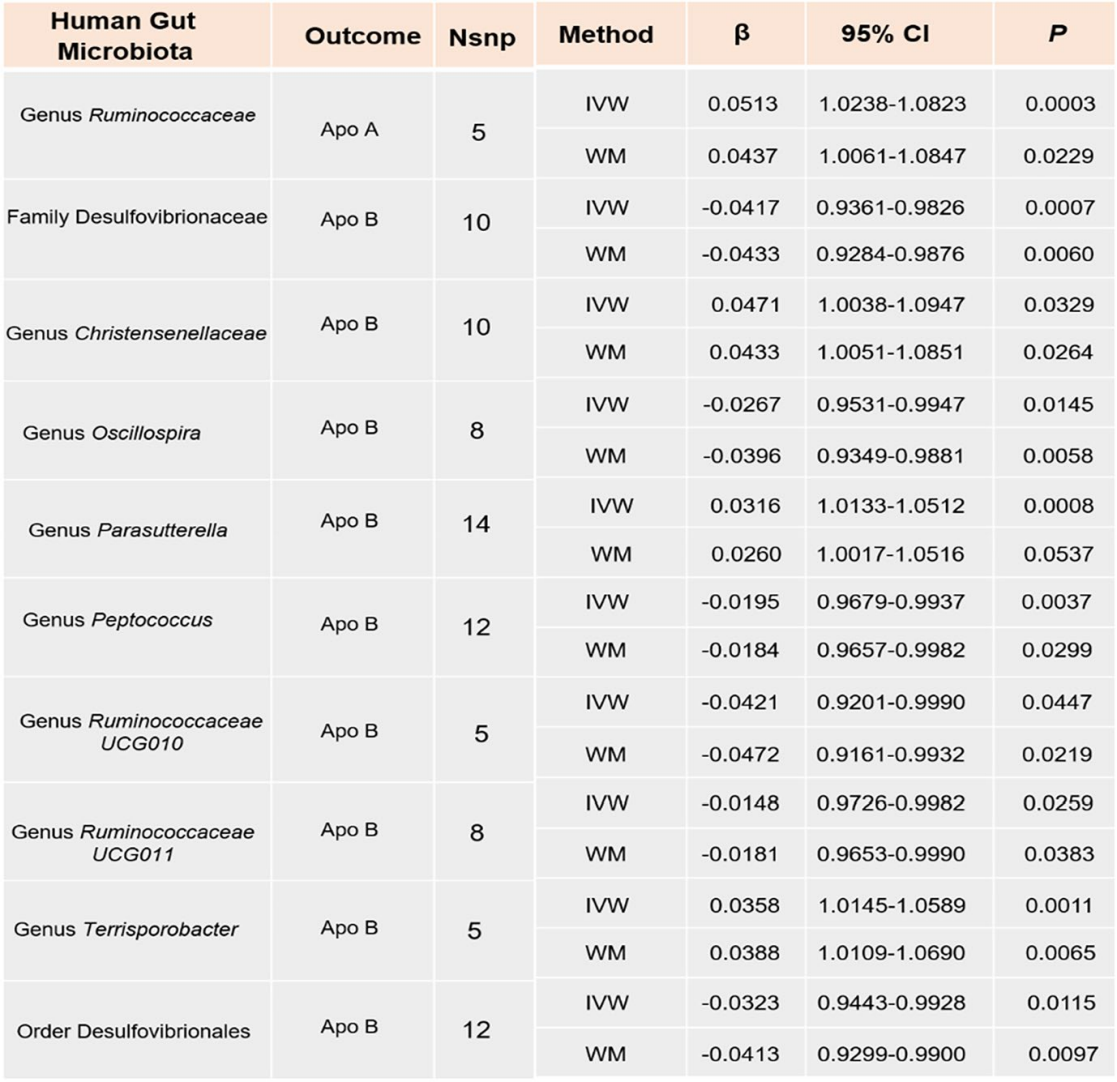
MR results of causal relationships between intestinal flora and both Apo A, and ApoB (*P*_IVW_ < 0.05 and *P*_WM_ < 0.05). *Nsnp* Number of the SNP used as the IVs for the MR analyses, *IVW* inverse variance weighted, *WM* Weighted median, CI Confidence interval

#### ApoB

We used the IVW method to screen twenty intestinal flora that were causally related to ApoB (*P* < 0.05) (Supplementary Table S2). To make the results more reliable, we obtained 9 stable results when considering WM analysis. As shown in Table 1, We found that the genus *Christensenellaceae* [Estimate = 0.0471, 95% confidence interval [CI]: 1.0038–1.0947, *P* = 0.0329], genus *Parasutterella* [Estimate = 0.0316, 95% confidence interval [CI]: 1.0133–1.0512, *P*= 0.0008], and genus *Terrisporobacter* [Estimate = 0.0358, 95% confidence interval [CI]: 1.0145–1.0589,  *P*= 0.0010] were positively correlated with the serum level of ApoB. Differently, family Desulfovibrionaceae [Estimate = -0.0418, 95% confidence interval [CI]: 0.9362–0.9826,  *P*= 0.0007], genus *Oscillospira* [Estimate = -0.0267, 95% confidence interval [CI]: 0.9531–0.9947, *P* = 0.0145], genus *Peptococcus* [Estimate = -0.0195, 95% confidence interval [CI]: 0.9679–0.9937, *P* = 0.0037], genus *Ruminococcaceae UCG010* [Estimate = -0.0421, 95% confidence interval [CI]: 0.9201–0.9990,  *P*= 0.0447], genus *Ruminococcaceae UCG011* [Estimate = -0.0148, 95% confidence interval [CI]: 0.9726–0.9982,  *P*= 0.0259], and order *Desulfovibrionales* [Estimate = -0.0323, 95% confidence interval [CI]: 0.9443–0.9928, *P*= 0.0115] showed a negative association with serum levels of ApoB. The scatter plots and forest plots for the analyses are shown in Supplementary Figs. 5–6.

Next, to further demonstrate the reliability of our results, we carried out a sensitivity analysis. As shown in Table 3, except for the genus *Christensenellaceae* group, no heterogeneity was found in the other groups (Cochrane’s Q test, *P* > 0.05). MR-Egger intercept test and MR-PRESSO global test suggest that there was no horizontal pleiotropy or outliers in all groups (*P* > 0.05).

#### LDL-C

By employing the IVW method, we identified causal associations between eleven intestinal flora and LDL-C (Supplementary Table S2). After integrated with WM method, three bacterial taxa were still stable. Our MR analysis found that the genus *Oscillospira* [Estimate = -0.0257, 95% confidence interval [CI]: 0.9519–0.9519, *P* = 0.0322] was considerably associated with lower LDL-C. While the genus *Parasutterella* [Estimate = 0.0254, 95% confidence interval [CI]: 1.0090–1.0427, *P* = 0.0025] and genus *Terrisporobacte*r [Estimate = 0.0364, 95% confidence interval [CI]: 1.0110–1.6038, *P* = 0.0050] were considerably associated with higher LDL-C (Table 2). The scatter plots and forest plots for the analyses are shown in Supplementary Figs. 9–10. Based on the results of the MR-Egger and MR-PRESSO tests, no horizontal pleiotropy or outliers were found (*P* > 0.05) (Table 3). No significant heterogeneity was found in the results of Cochrane's Q test (*P* > 0.05).

**Table 2.**
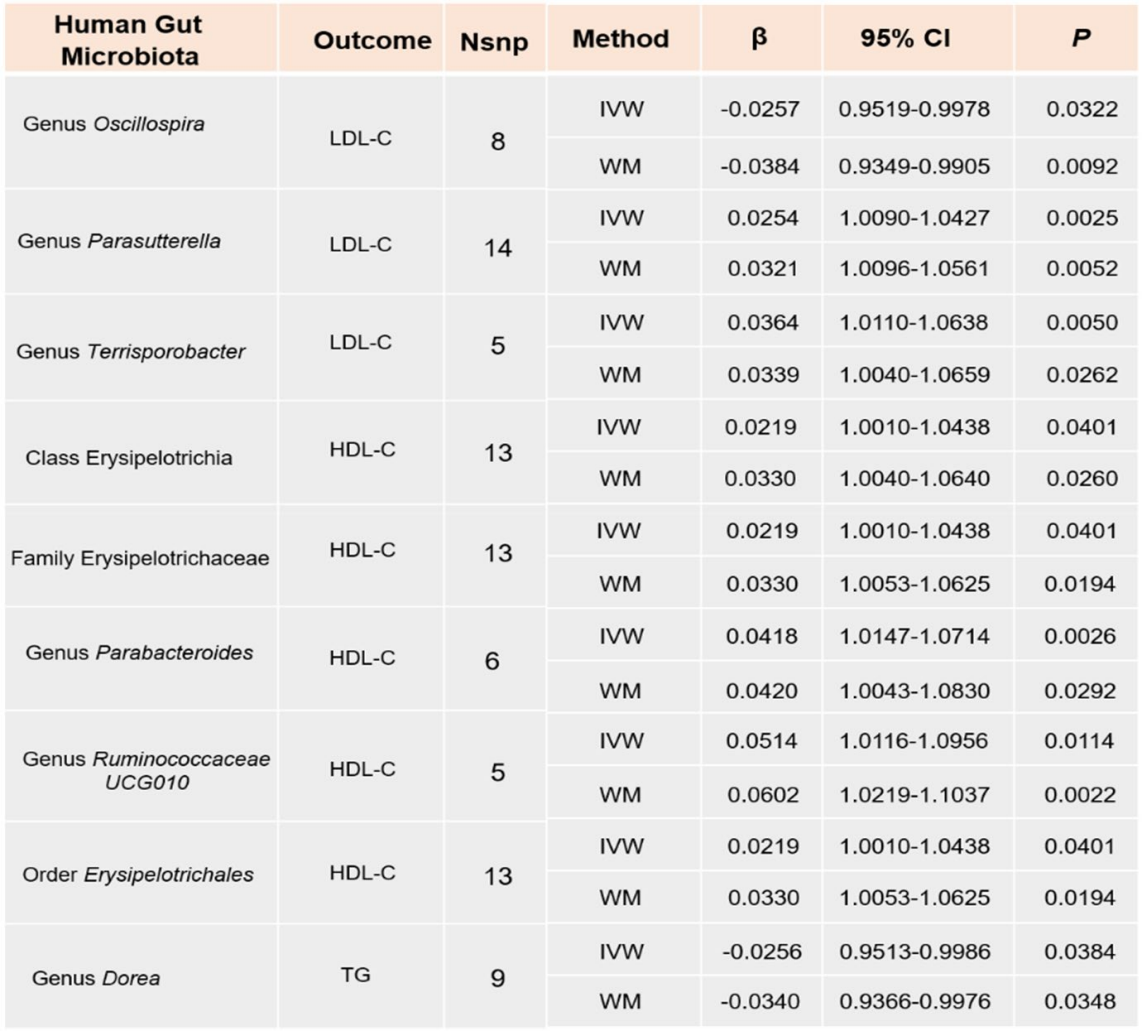
MR results of causal relationships between intestinal flora and LDL-C, HDL-C, and TG (*P*_IVW_ < 0.05 and *P*_WM_ < 0.05). *Nsnp* Number of the SNP used as the IVs for the MR analyses, *IVW* Inverse variance weighted, *WM* Weighted median, *CI* Confidence interval

**Table 3.**
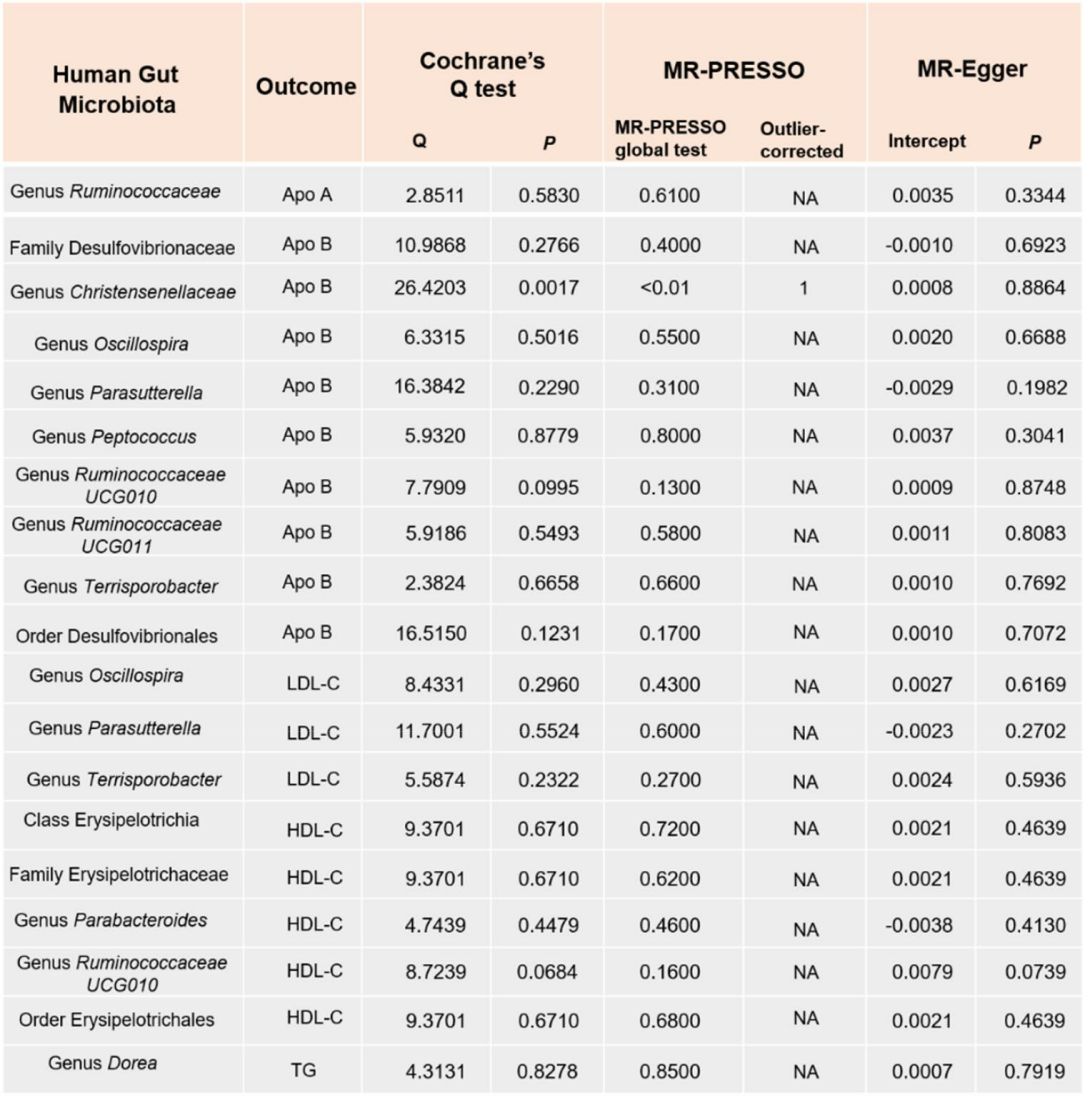
Results of heterogeneity and horizontal pleiotropy

#### HDL-C

The IVW method revealed a causal relationship between twenty-one intestinal flora and HDL-C (Supplementary Table S2). However, after considering WM method, only five bacterial taxa remained stable. In specific, class Erysipelotrichia [Estimate = 0.0219, 95% confidence interval [CI]: 1.0090–1.0427, *P* = 0.0401], family Erysipelotrichaceae [Estimate = 0.0219, 95% confidence interval [CI]: 1.0090–1.0427, *P* = 0.0401], genus *Parabacteroides* [Estimate = 0.0418, 95% confidence interval [CI]: 1.0090–1.0427, *P* = 0.0026], genus *Ruminococcaceae*[Estimate = 0.0514, 95% confidence interval [CI]: 1.0090–1.0427, *P* = 0.0114], and order Erysipelotrichales [Estimate = 0.0219, 95% confidence interval [CI]: 1.0090–1.0427, *P* = 0.0401] were considerably associated with higher HDL-C (Table 2). The scatter plots and forest plots for the analyses are shown in Supplementary Figs. 13–14. The results of the MR-Egger and MR-PRESSO tests confirmed that there was no horizontal pleiotropy (*P* > 0.05) and the outcomes from Cochrane’s Q test demonstrated that there was no obvious heterogeneity among the selected SNPs (*P* > 0.05).

#### TG

Using the IVW method, we preliminarily screened six intestinal flora associated with TG (Supplementary Table S2). Only one bacterial taxon remained stable after WM method validation. Specifically speaking, a higher genetically predicted *genus Dorea* [Estimate = -0.0256, 95% confidence interval [CI]: 0.9513–0.9986, *P* = 0.0384] was associated with a lower level of TG (Table 2). No significant heterogeneity or horizontal pleiotropy was found based on the results of Cochrane's Q, MR-Egger, and MR-PRESSO tests (*P* > 0.05) (Table 3).

### Bonferroni-corrected test and sensitivity analysis

**Fig. 2:**
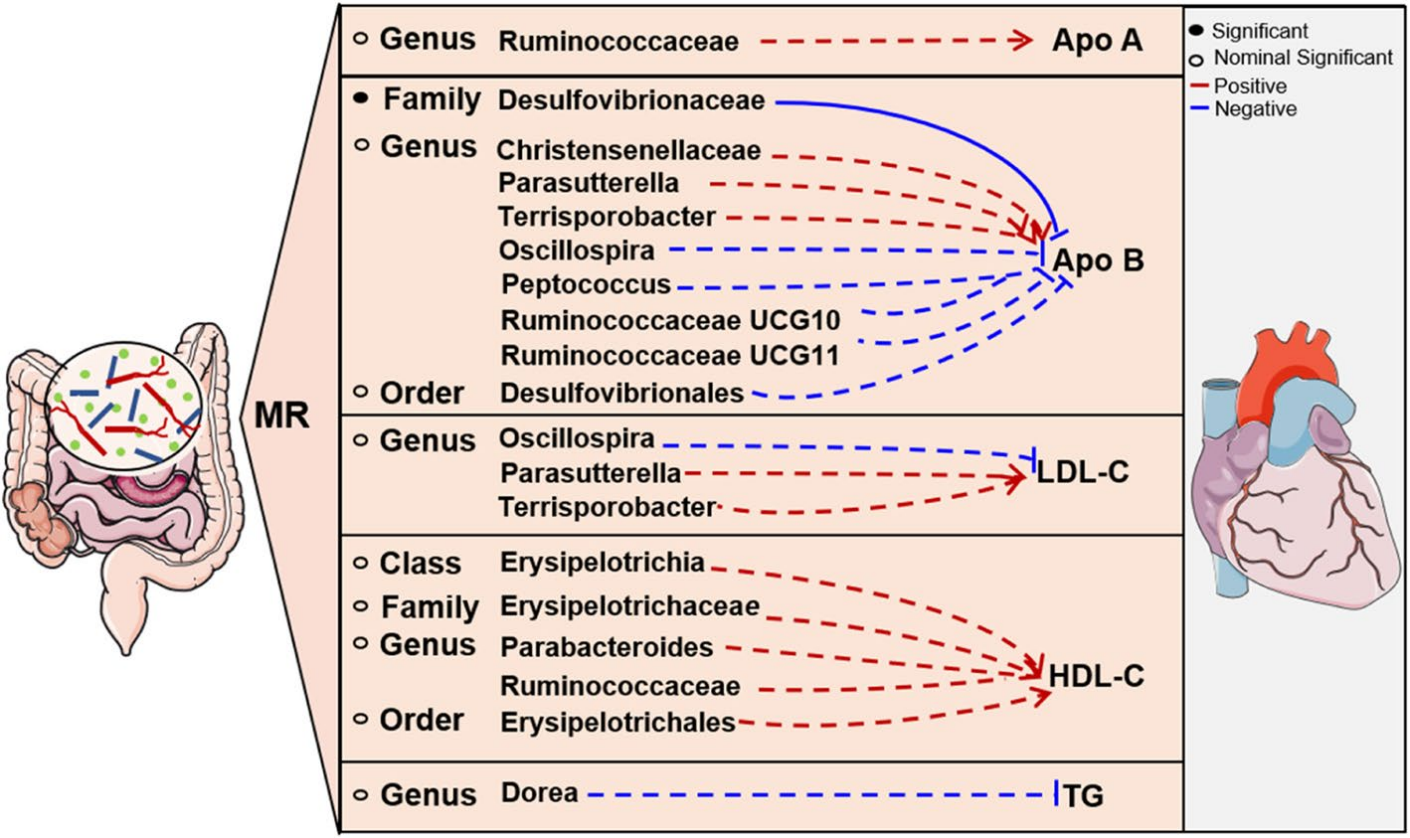
Significant and nominal links between intestinal flora and lipids

After the Bonferroni-correction test, only Desulfovibrionaceae falls below the Bonferroni-corrected threshold (Supplementary Table S6). This indicates that higher levels of Desulfovibrionaceae [Estimate = -0.0418, 95% confidence interval [CI]: 0.9362–0.9826, *P* = 0.0007] still exhibit a more significant and stable inverse causality with serum ApoB levels. Figure 2 shows significant and nominal links between intestinal flora and lipids.

Leave-one-out analysis is a step-by-step process of removing each SNP and observing whether the results change after removing each SNP. Based on our findings, while the overall stability is observed, it appears that certain SNPs may exert a dominant influence on the positive outcomes (Supplement Figure S3,7,11,15,19). Additionally, to prevent reverse causality from interfering with the above results, we performed a reverse MR analysis with significant intestinal flora in two-sample MR studies as the outcome and lipids as the exposure on locus-wide significance level (Supplementary Table S7). There was no evidence for a causal effect of lipids on the intestinal flora.

## Discussion

In the past decade, our comprehension of the intestinal flora has undergone a nearly exponential expansion [40]. The increasing recognition of the importance of the intestinal flora is helped by the advent of innovative methodologies and technologies, including germ-free animals [41], fecal microbiota transplantation [42], and omics [43]. Genetic studies have estimated that human genetics can explain 1.9%-8.1% of the variation in the gut microbiome [44, 45]. Some of these variants might be associated with certain traits, such as inflammatory bowel disease [46] and tumors [47]. Dyslipidemia is a significant risk factor for CVD. Recent investigations suggest a potential influence of the intestinal flora on circulating lipid levels. On this basis, we employed the large, publicly available GWAS database and applied MR analysis to explore the causal relationship between intestinal flora and lipids [24, 25]. In the present study, we identified a total of 19 lipid-related intestinal flora. Among them, a significant negative causal relationship exists between Desulfovibrionaceae and ApoB. Besides, no reverse causality was found by the reverse MR analysis.

Desulfovibrionaceae is an important anaerobic bacterium in the digestive tract. It has the capability to bind with human colonic mucin and is enriched on the mucosal surface of the colon [48, 49]. Researchers have noted a negative correlation between *Desulfovibrio* and obesity indicators such as BMI [50] and waist [51]. An important characteristic of *Desulfovibrio* is its ability to perform dissimilatory sulfate reduction by utilizing sulfate as an electron acceptor for respiration, thereby producing hydrogen sulfide (H_2_S) [52]. As an important gas transmitter, H_2_S is involved in numerous biological processes, including posttranslational modifications of proteins by S-sulfhydration in the cardiovascular system [53] and lipid metabolism [54]. Some studies indicate that the reduction of H_2_S is associated with an accelerated occurrence of atherosclerosis [55, 56]. After feeding Cystathionine γ-lyase-deficient mice to a high-fat diet for 12 weeks, Mani observed significant disturbances in lipid metabolism and early atheromatous changes in the aorta. Treatment of these animals with the rapid H_2_S donor sodium hydrosulfide reduced the development of atherosclerosis [55].

This may suggest that future interventions on H_2_S could potentially serve as a viable direction for maintaining lipid metabolism homeostasis and slowing the development of atherosclerosis. However, at the current stage, how to manipulate H_2_S levels in a physiologically appropriate manner is a major concern. Desulfovibrionaceae as an important endogenous source of H_2_S, or targeting of Desulfovibrionaceae will help future studies in this regard.

Interestingly, metagenomics revealed that *Desulfovibrio* can produce acetic acid [57, 58], which, as an important member of short-chain fatty acids (SCFAS), is undoubtedly essential for lipid metabolism homeostasis [59]. Acetic acid can activate the AMP-activated protein kinase signaling pathway to regulate hepatic lipid metabolism [60]. Moreover, the polymorphism of gut microbial communities, particularly those associated with lipid metabolic homeostasis, such as *Coprococcus*, *Ruminococcus*, *Akkermansia*, *Roseburia*, and *Faecalibacterium*, closely correlates with the relative abundance of Desulfovibrionaceae. The protective effects of *Coprococcus* [61], *Ruminococcus *[62], *Akkermansia *[63], *Roseburia *[64], and *Faecalibacterium*[65] are associated with the production of SCFAS. This phenomenon could have a synergistic effect with acetic acid produced by Desulfovibrionaceae, contributing to the maintenance of lipid metabolism homeostasis and the protection of host health. It is imperative to acknowledge that, while these mechanisms provide initial insights into the association between Desulfovibrionaceae and blood lipids, further investigation is still needed for a comprehensive understanding of the specific underlying mechanisms.

Additionally, ApoB functions as the primary transporter of LDL-C, and these two components are intricately connected within the organism. Elevated levels of LDL-C unquestionably expedite the progression of atherosclerosis, and our study indicates that certain intestinal flora may synergistically affect both. *Oscillospira*, an intestinal anaerobe, can utilize host glycans and produce butyrate [66]. Butyrate plays a crucial role in maintaining metabolic homeostasis [67]. In animal models of metabolic diseases, supplementation with butyrate reportedly confers numerous benefits, including reduced serum triglycerides, total cholesterol and glucose, and reduced weight gain in response to a high fat diet (HFD) [68–70] This protective effect may be attributed to epigenetic effects through inhibition of histone deacetylases (HDACs). HDACs are a group of epigenetic modifying enzymes that remove acetyl groups from histone tails, thereby modifying chromatin structure and the accessibility of genes for transcription [71]. HDACs regulate a variety of metabolic pathways and deregulation of HDACs has been associated with CVD [72]. Apart from this. Butyrate can bind and activate the G protein-coupled (GPR) free fatty acid receptors (FFAR) [73], influencing the release of gut hormones. These gut hormones may play an important role in appetite suppression and lipid metabolism [74]. In our study, *Parasutterella* also could affect both Apo B and LDL-C. In a study on obesity, researchers found that *Parasutterella* could impact human fatty acid synthesis [75]. This may exert a direct impact on ApoB production and LDL-C metabolism. *Parasutterella* colonies were also found to be significantly enriched in mice susceptible to obesity [76]. Future interventions targeting *Parasutterella* may be a feasible way to combat obesity and maintain lipid homeostasis. Apart from this, our analysis complements the findings of Lee. Lee et al. found that *Terrisporobacter* could affect TG and HDL-C [77], We will further delineate the causal relationship between *Terrisporobacter* and ApoB and LDL-C. We are confident that our study can establish a more solid research foundation for future investigations.

In addition to the "bad cholesterol" mentioned above, HDL-C is widely recognized as the "good cholesterol" in our circulation. The latest research indicates that with each unit increase in HDL-C level, there is a corresponding 2–3% reduction in the risk of CVD [78]. In the present MR analysis, we find a positive causal relationship between some intestinal flora and HDL-C, such as *Erysipelotrichia*. *Erysipelotrichia* is an important bacterium for maintaining intestinal health. *Erysipelotrichia* microflora transplantation has demonstrated great potential advantages in promoting intestinal regeneration after radiation [79, 80]. The crucial ability to maintain intestinal health is poised to become a significant consideration in the treatment of chronic diseases such as atherosclerosis in the future. Our results also suggest that *Ruminococcaceae* affects lipid metabolism. Priscilla et al. had observed a significant increase in the abundance of *Ruminococcaceae* in the control group compared to patients with atherosclerotic dyslipidemia [81]. According to our analysis, this increase in abundance may regulate apolipoprotein and cholesterol, consequently exerting a protective effect on the host. To our surprise, we find for the first time a potential link between *Dorea* and TG. *Dorea* [82] is a member of the family Lachnospiraceae which is reported to be strongly associated with lower TG levels in European and Chinese populations [83, 84]. Our study suggests that we cannot exclude the influence of *Dorea* on TG in this context, and we believe that our results can provide new evidence and confidence for the increasing of intestinal Dorea number in patients with dyslipidemia in the future.

In our study, although we did not observe a significant potential impact of blood lipids on the gut microbiota, it is important to note that certain genetic variations, such as the APOB rs693, may serve as an independent risk for dyslipidemia [85]. In this subset of patients, the importance of lipids on intestinal flora needs to be further elucidated to formulate individualized treatment plans.

We also need to acknowledge certain limitations in our study. Firstly, this study mainly included individuals of European ancestry, and additional validation is required when extending the results to other populations. Secondly, exposure factors such as diet and environment also have an impact on the composition and abundance of intestinal flora, we will treat it as the focus of our upcoming study. Lastly, despite the theoretical causal impact of certain bacterial groups, the specific mechanisms remain unclear. To elucidate the role of intestinal flora and its contribution to lipid homeostasis, both single flora transplantation and a substantial number of animal experiments are warranted. Our research team is currently engaged in related investigations to identify potential strategic targets for lipid level control.

In conclusion, our study examined the causal relationship between 211 intestinal flora and blood lipids. We screened 19 intestinal flora that might have an association with dyslipidemia in humans. Among them, Desulfovibrionaceae showed a stable and significant negative association with ApoB levels. These findings will provide a meaningful reference to discover dyslipidemia for intervention to address CVD in the clinic.

### Supplementary Information

**Supplementary Material 1. **

## Data Availability

Only publicly available GWAS summary data were used in this work. All raw data for this study are publicly available in the IEU Open GWAS Project repository (IEU OpenGWAS project (mercies. ac. UK)). Exposure dataset from MiBioGen consortium (https://mibiogen.gcc.rug.nl/). 211 GM taxa (including nine phyla, 16 classes, 20 orders, 35 families, and 131 genera). Outcome dataset can be found here: (https://mibiogen.gcc.rug.nl/UKBiobank.0
