# Supplementary material for: Causality of the gut microbiome and atherosclerosis-related lipids: a bidirectional Mendelian Randomization study
Source: BMC Cardiovasc Disord. 2024 Mar 2;24:138. doi: 10.1186/s12872-024-03804-3 (PMC10909291; doi:10.1186/s12872-024-03804-3)

Supplementary Figures

Causality of the gut microbiome and atherosclerosis-related lipids: A bidirectional Mendelian randomization study

Da Teng[1,2] †;Wenjuan Jia[1,2] †; Wenlong Wang[1,2]; Lanlan Liao[4]; Bowen Xu[3]; Lei Gong[1]; Haibin Dong[1]; Lin Zhong[1]*; Jun Yang[1]*

[1] Yantai Yuhuangding Hospital affiliated to Qingdao University, Yantai, Shandong, People's Republic of China.
[2] Qingdao University, Qingdao, Shandong, People's Republic of China.
[3] Binzhou Medical University, Yantai, Shandong, People's Republic of China.
[4] Dazhou Central Hospital, Dazhou, Sichuan, People's Republic of China.
† These authors have contributed equally to this work and share the first authorship

Correspondence**:**
Jun Yang:: yangjyhd@163.com Lin Zhong:: yizun1971@126.com

Supplement FigureS1

Scatter plots of nominal significant estimates from genetically predicted microbiotas ‘genus Ruminococcaceae ’on ‘ApoA’

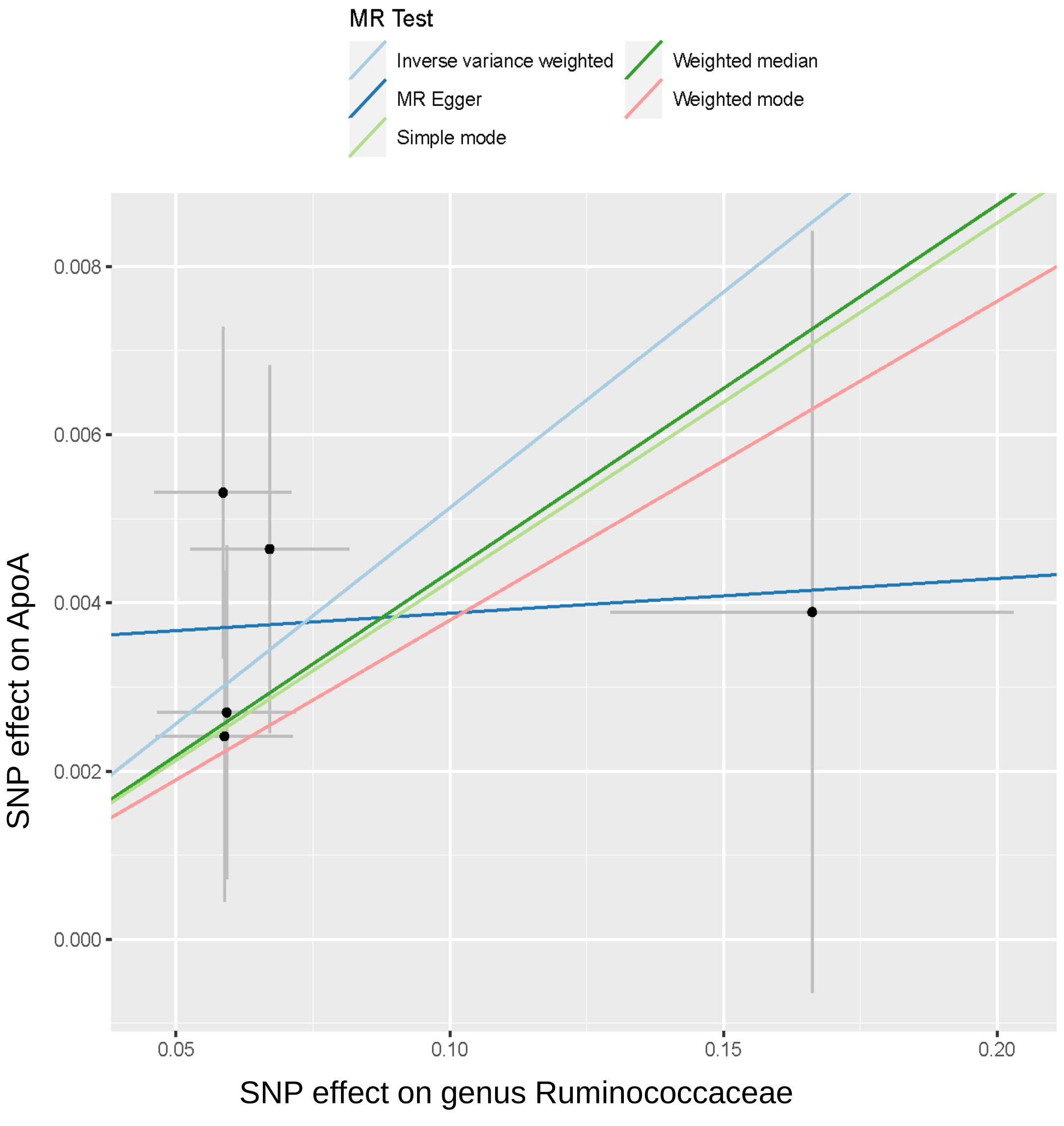

Supplement FigureS2

Forest plots of nominal significant estimates from genetically predicted microbiotas ‘genus Ruminococcaceae ’on ‘ApoA’

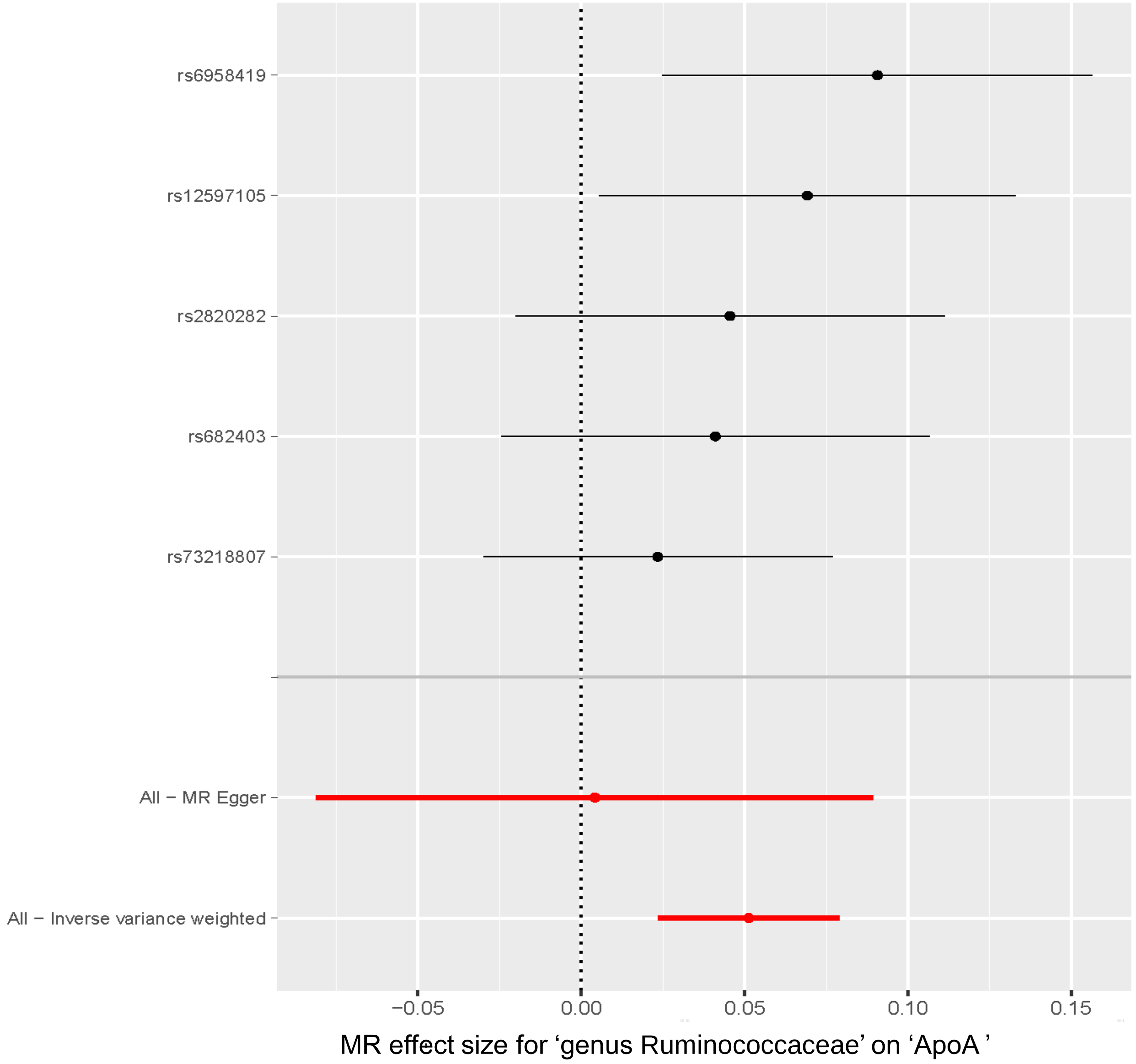

Supplement FigureS3
Leave-one-out plots of nominal significant estimates from genetically predicted microbiotas ‘genus Ruminococcaceae ’on ‘ApoA’

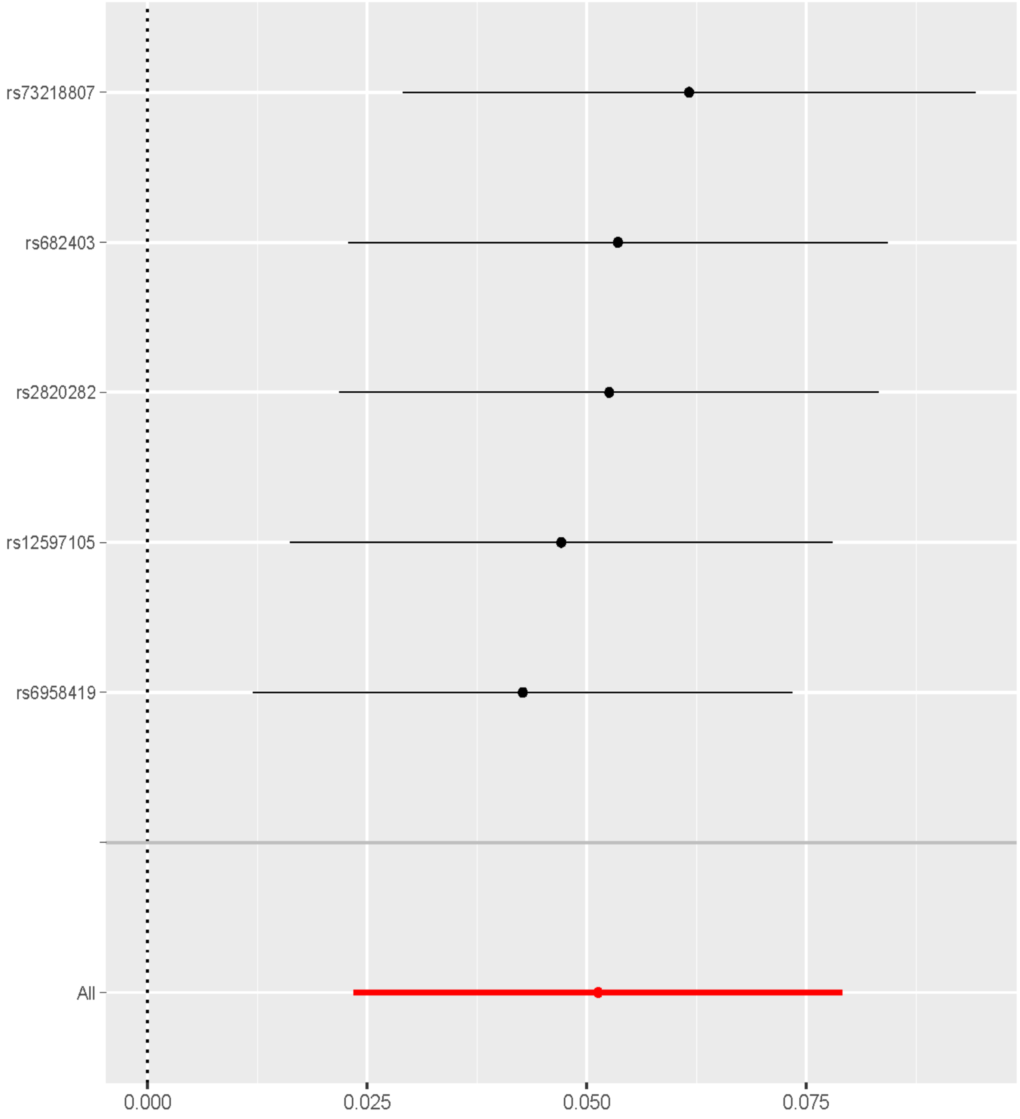

MR Leave-one-out sensitivity for ‘genus Ruminococcaceae’ on ‘ApoA ’

Supplement FigureS4
Funnel plots of nominal significant estimates from genetically predicted microbiotas ‘genus Ruminococcaceae ’on ‘ApoA’

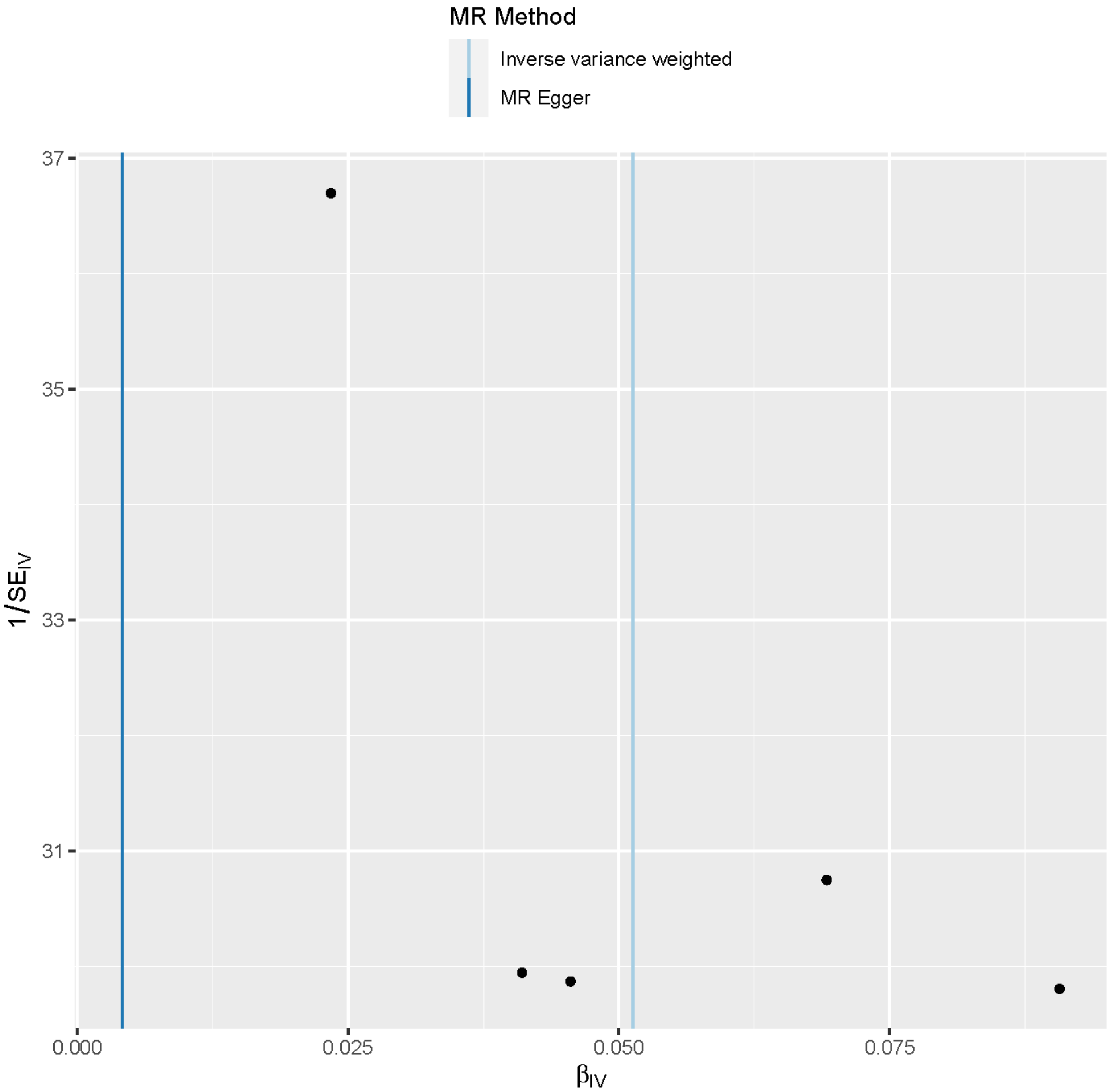

Supplement FigureS5

Scatter plots of nominal significant estimates from genetically predicted microbiotas{

A:Family Desulfovibrionaceae;

B:genus Christensenellaceae;

C:genus Oscillospira;

D:genus Parasutterella;

E:genus Peptococcus;

F:genus Ruminococcaceae UCG010;

G:genus Ruminococcaceae UCG011;

H:genus Terrisporobacter;

I:order Desulfovibrionales} on ApoB

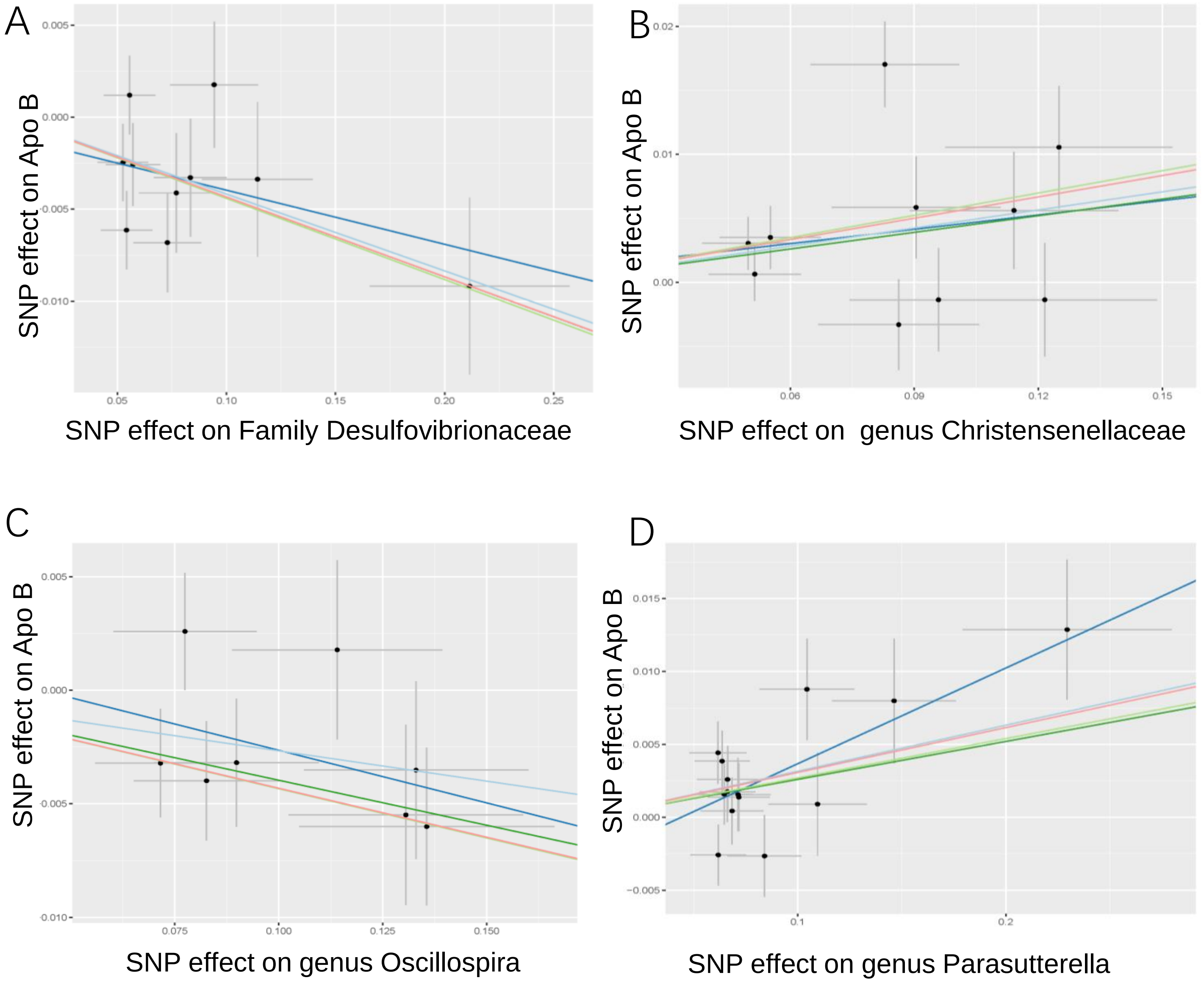

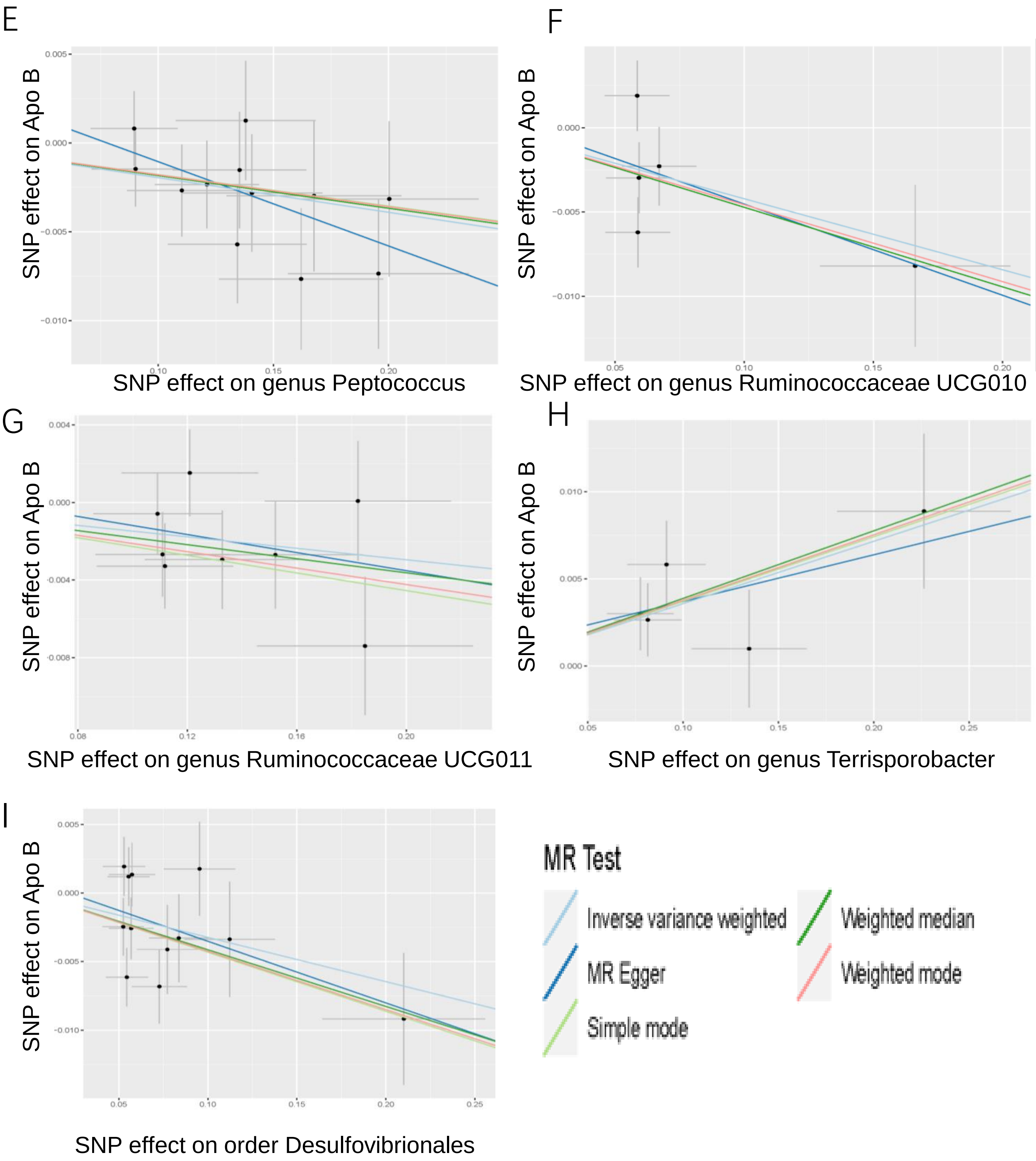
E
SNP effect on Apo B
SNP effect on genus Peptococcus
F
SNP effect on Apo B
SNP effect on genus Ruminococcaceae UCG010
G
SNP effect on Apo B
SNP effect on genus Ruminococcaceae UCG011
H
SNP effect on Apo B
SNP effect on genus Terrisporobacter
I
SNP effect on Apo B
SNP effect on order Desulfovibrionales
MR Test
Inverse variance weighted
MR Egger
Simple mode
Weighted median
Weighted mode

Supplement FigureS6

Forest plots of nominal significant estimates from genetically predicted microbiotas{
A:Family Desulfovibrionaceae;
B:genus Christensenellaceae;
C:genus Oscillospira;
D:genus Parasutterella;
E:genus Peptococcus;
F:genus Ruminococcaceae UCG010;
G:genus Ruminococcaceae UCG011;
H:genus Terrisporobacter;
 I:order Desulfovibrionales} on ApoB

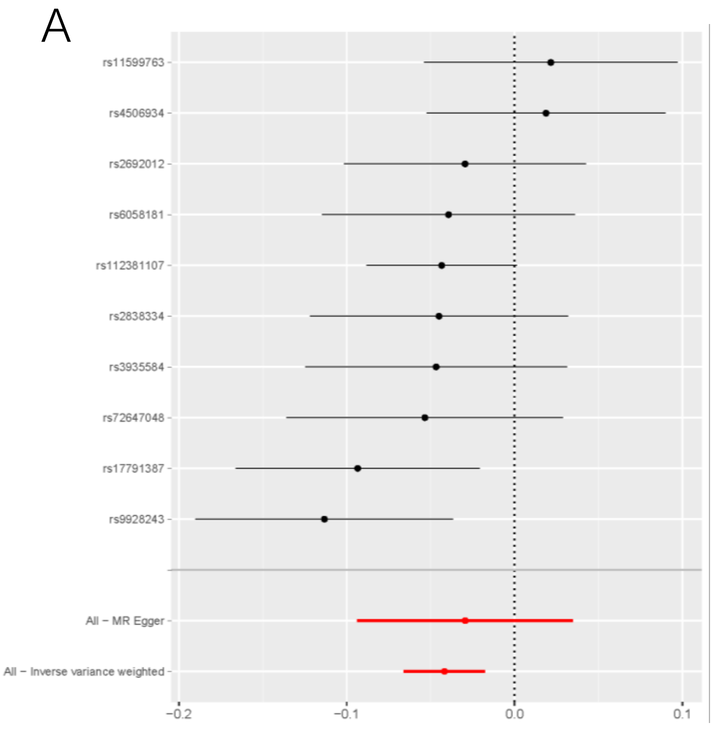

MR effect size for ‘Family Desulfovibrionaceae’ on ‘ApoB ’

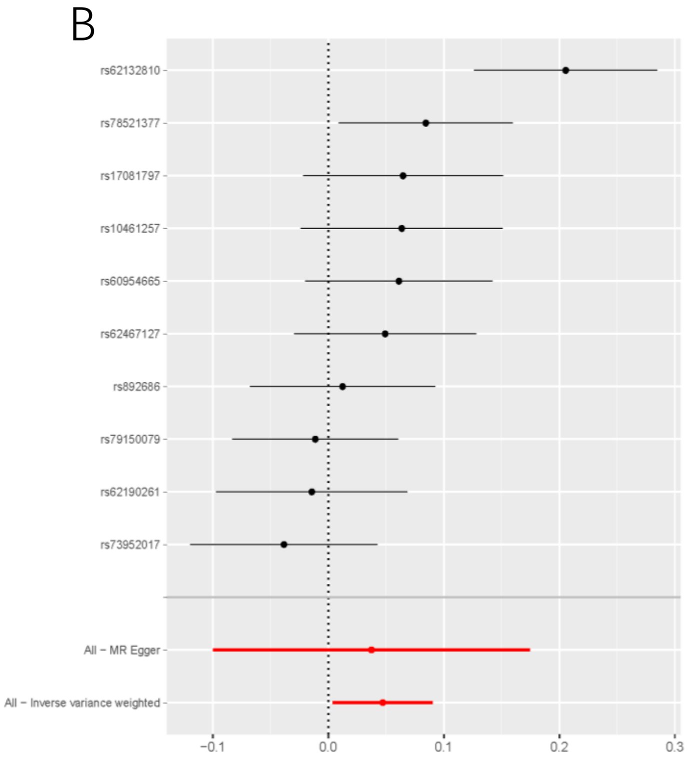

MR effect size for ‘genus Christensenellaceae’ on ‘ApoB ’

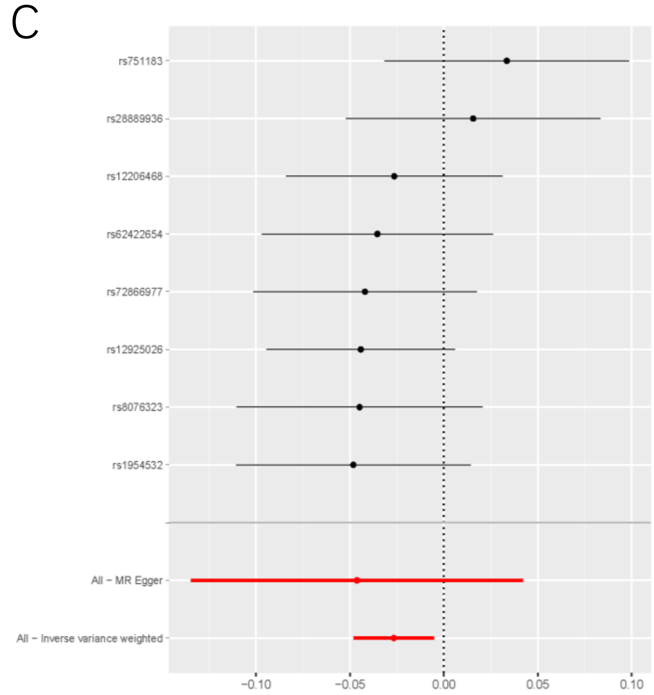

MR effect size for ‘genus Oscillospira’ on ‘ApoB ’

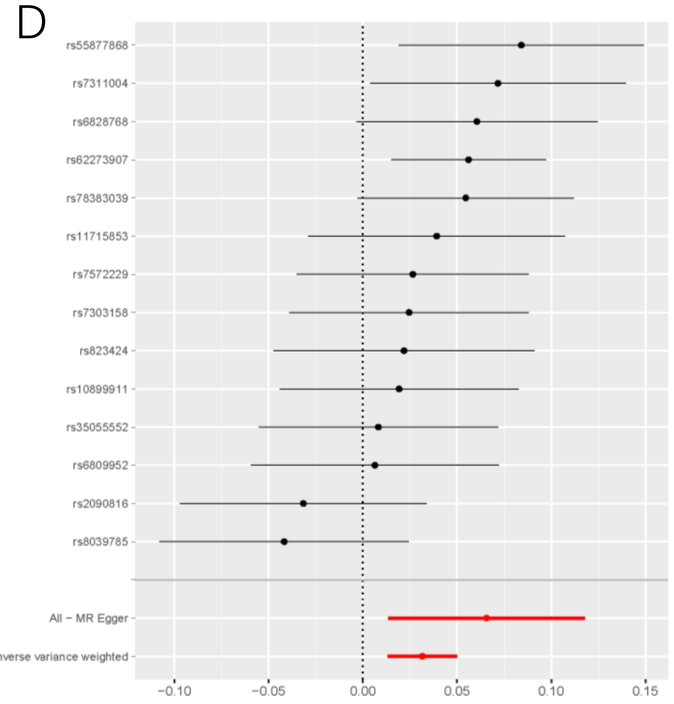

MR effect size for ‘genus Parasutterella’ on ‘ApoB ’

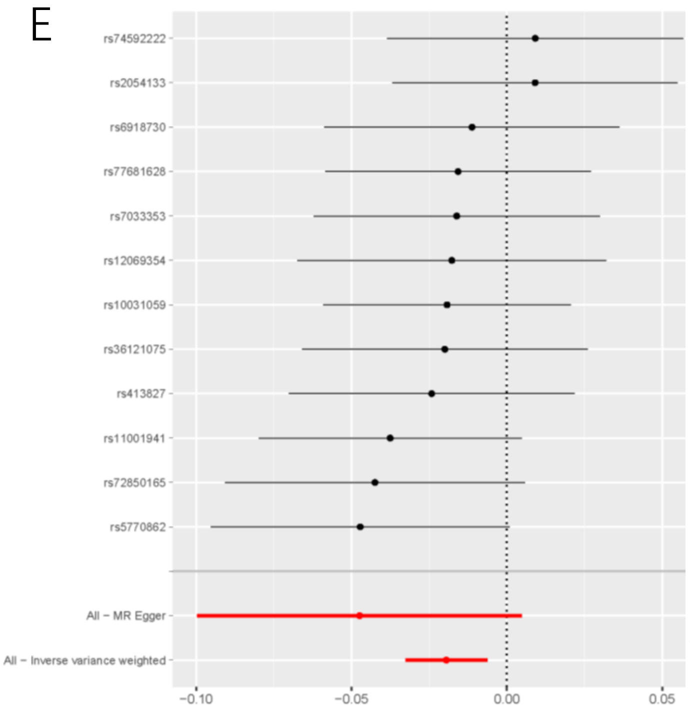

MR effect size for ‘genus Peptococcus’ on ‘ApoB ’

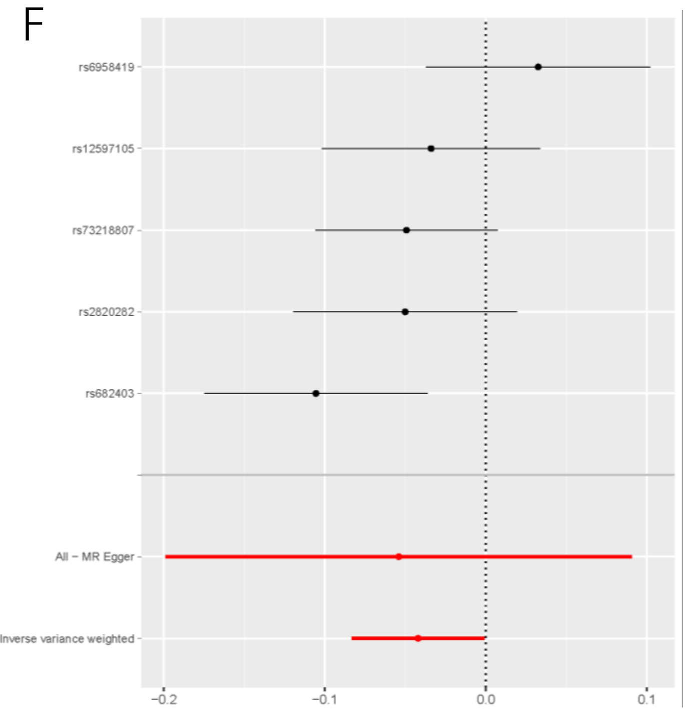

MR effect size for ‘genus Ruminococcaceae UCG010’ on ‘ApoB ’

G

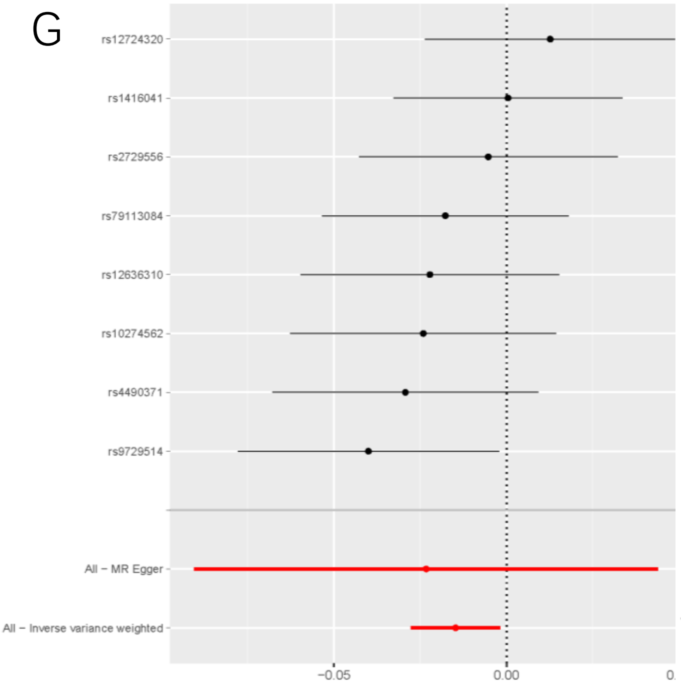

MR effect size for ‘genus Ruminococcaceae UCG011’ on ‘ApoB ’

H

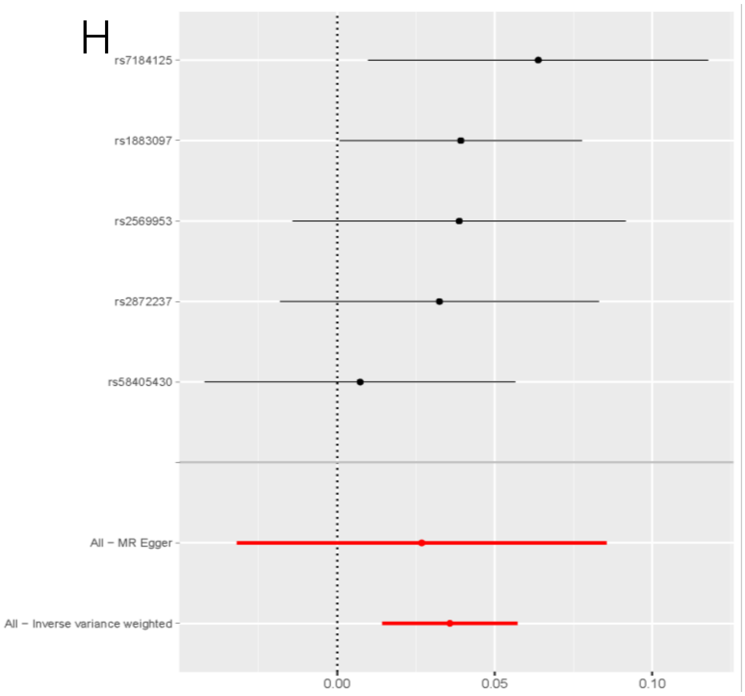

MR effect size for ‘genus Terrisporobacter’ on ‘ApoB ’

I

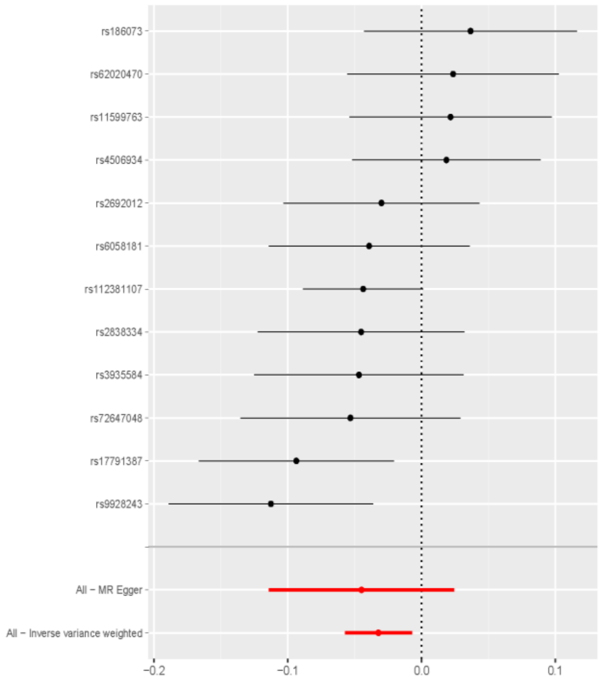

MR effect size for ‘order Desulfovibrionales’ on ‘ApoB ’

Supplement FigureS7

Leave-one-out plots of nominal significant estimates from genetically predicted microbiotas {

A: Family Desulfovibrionaceae;

B: genus Christensenellaceae;

C:genus Oscillospira;

D:genus Parasutterella;

E:genus Peptococcus;

F:genus Ruminococcaceae UCG010;

G:genus Ruminococcaceae UCG011;

H:genus Terrisporobacter;

I:order Desulfovibrionales} on ApoB

A

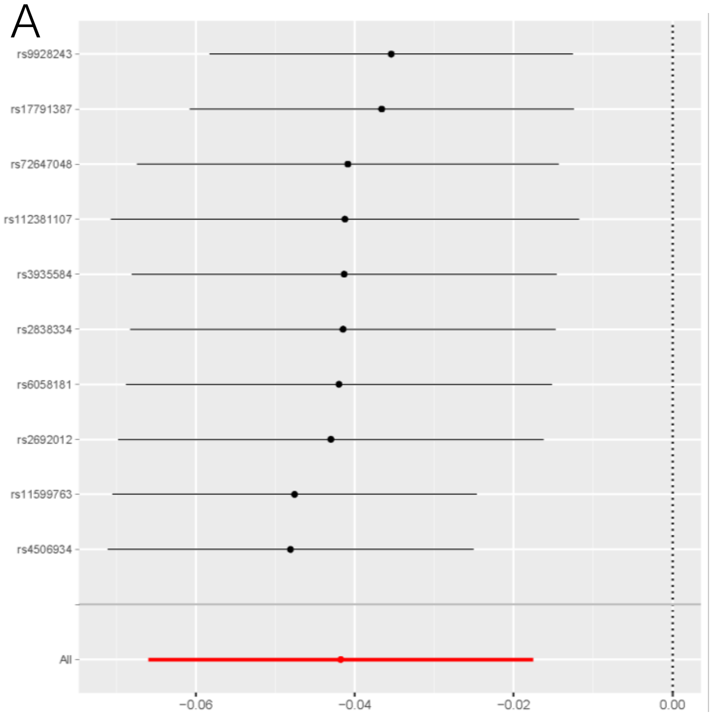

MR Leave-one-out sensitivity for ‘Family Desulfovibrionaceae’ on ‘ApoB’

B

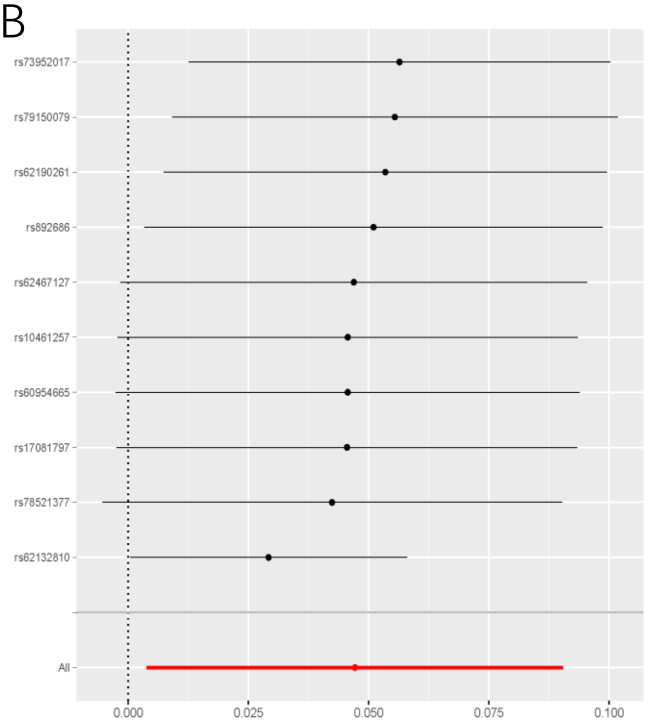

MR Leave-one-out sensitivity for genus Christensenellaceae’ on ‘ApoB’

C

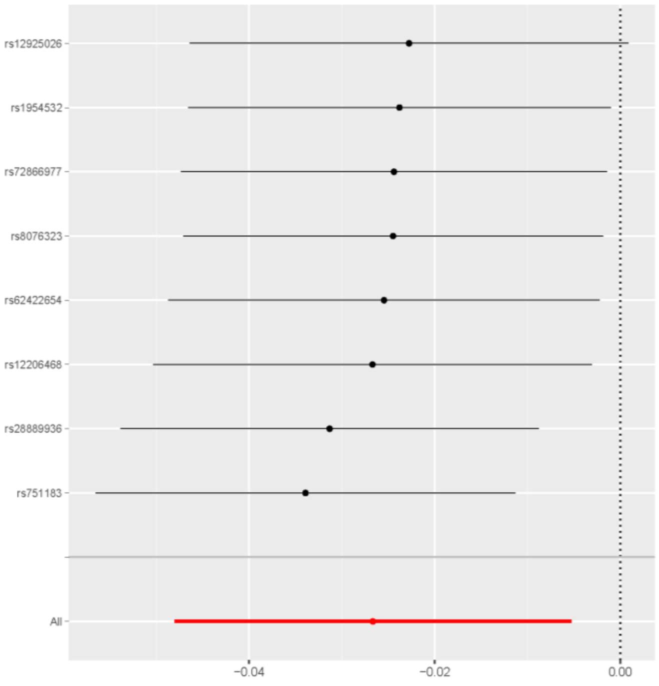

MR Leave-one-out sensitivity for ‘genus Oscillospira’ on ‘ApoB’

D

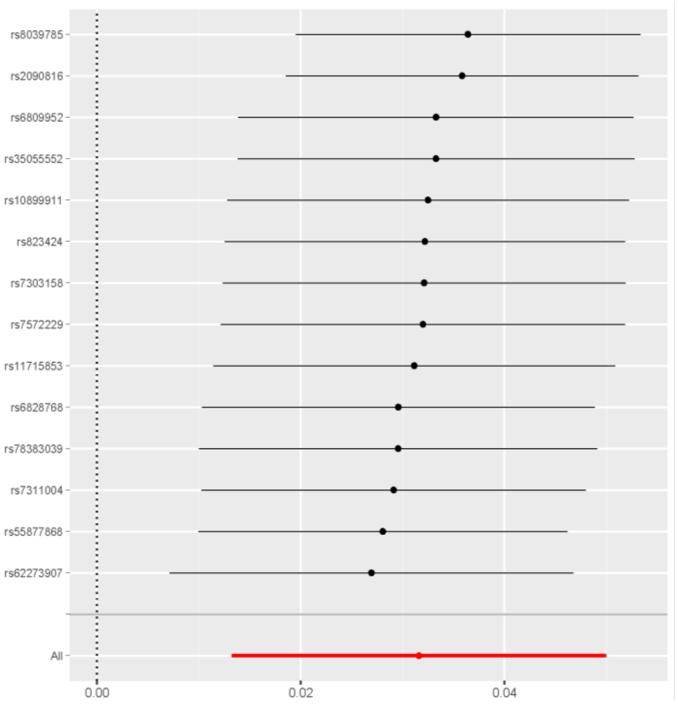

MR Leave-one-out sensitivity for ‘genus Parasutterella’ on ‘ApoB’

E

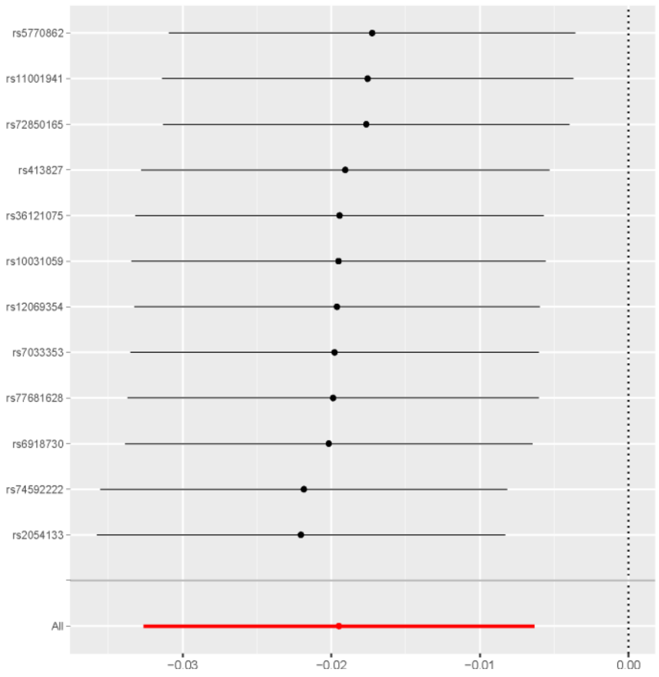

MR Leave-one-out sensitivity for ‘genus Peptococcus’ on ‘ApoB’

F

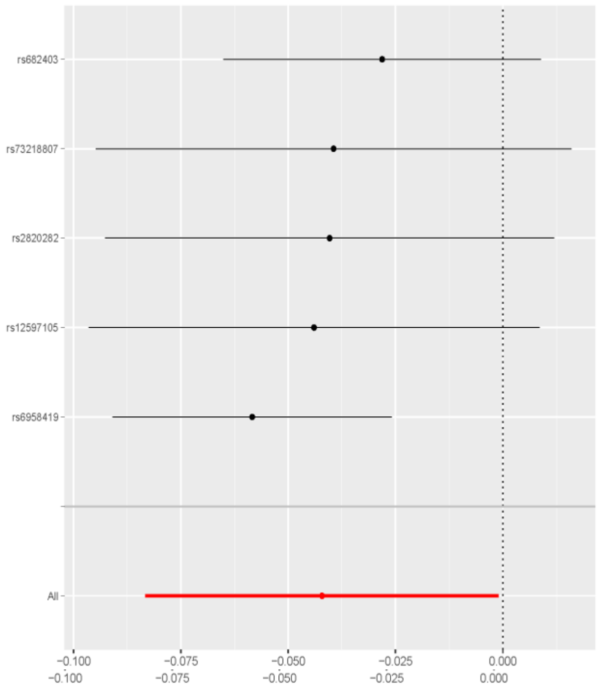

MR Leave-one-out sensitivity for ‘genus Ruminococcaceae UCG010 ’ on ‘ApoB’

G

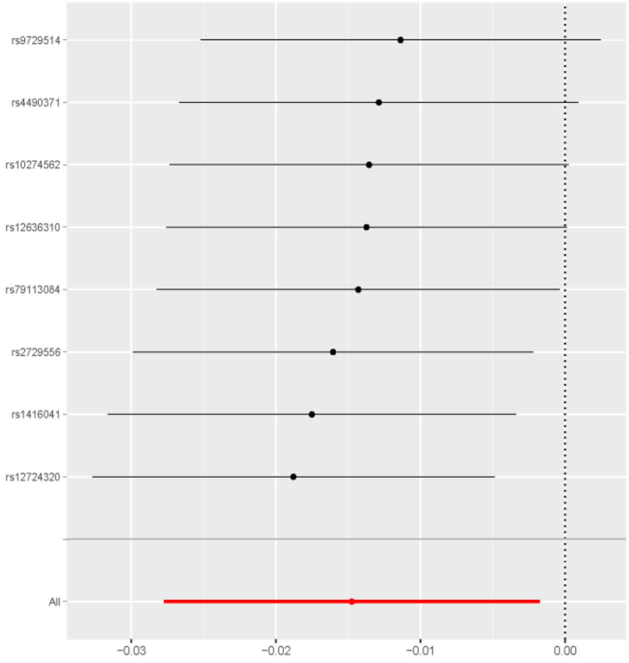

MR Leave-one-out sensitivity for ‘genus Ruminococcaceae UCG011’ on ‘ApoB’

H

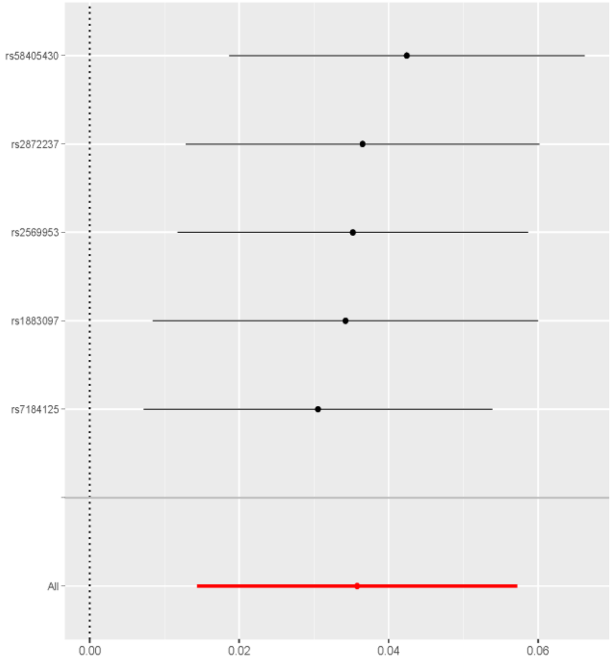

MR Leave-one-out sensitivity for ‘genus Terrisporobacter’ on ‘ApoB’

I

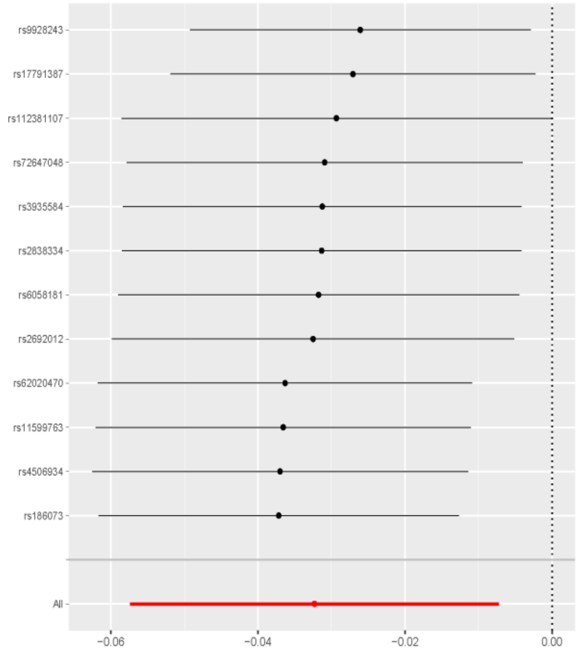

MR Leave-one-out sensitivity for ‘order Desulfovibrionales’ on ‘ApoB’

Supplement FigureS8

Funnel plots of nominal significant estimates from genetically predicted microbiotas{

A: Family Desulfovibrionaceae;

B: genus Christensenellaceae;

C:genus Oscillospira;

D:genus Parasutterella;

E:genus Peptococcus;

F:genus Ruminococcaceae UCG010;

G:genus Ruminococcaceae UCG011;

H:genus Terrisporobacter;

I: order Desulfovibrionales} on ApoB

A

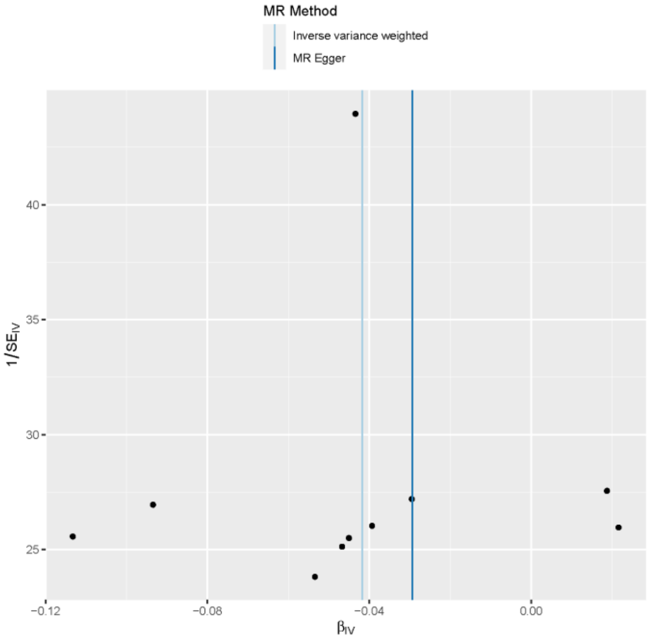

B

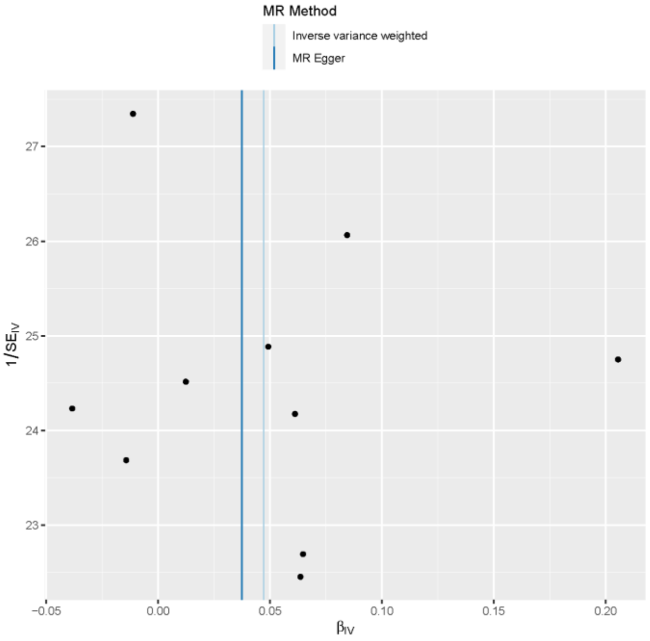

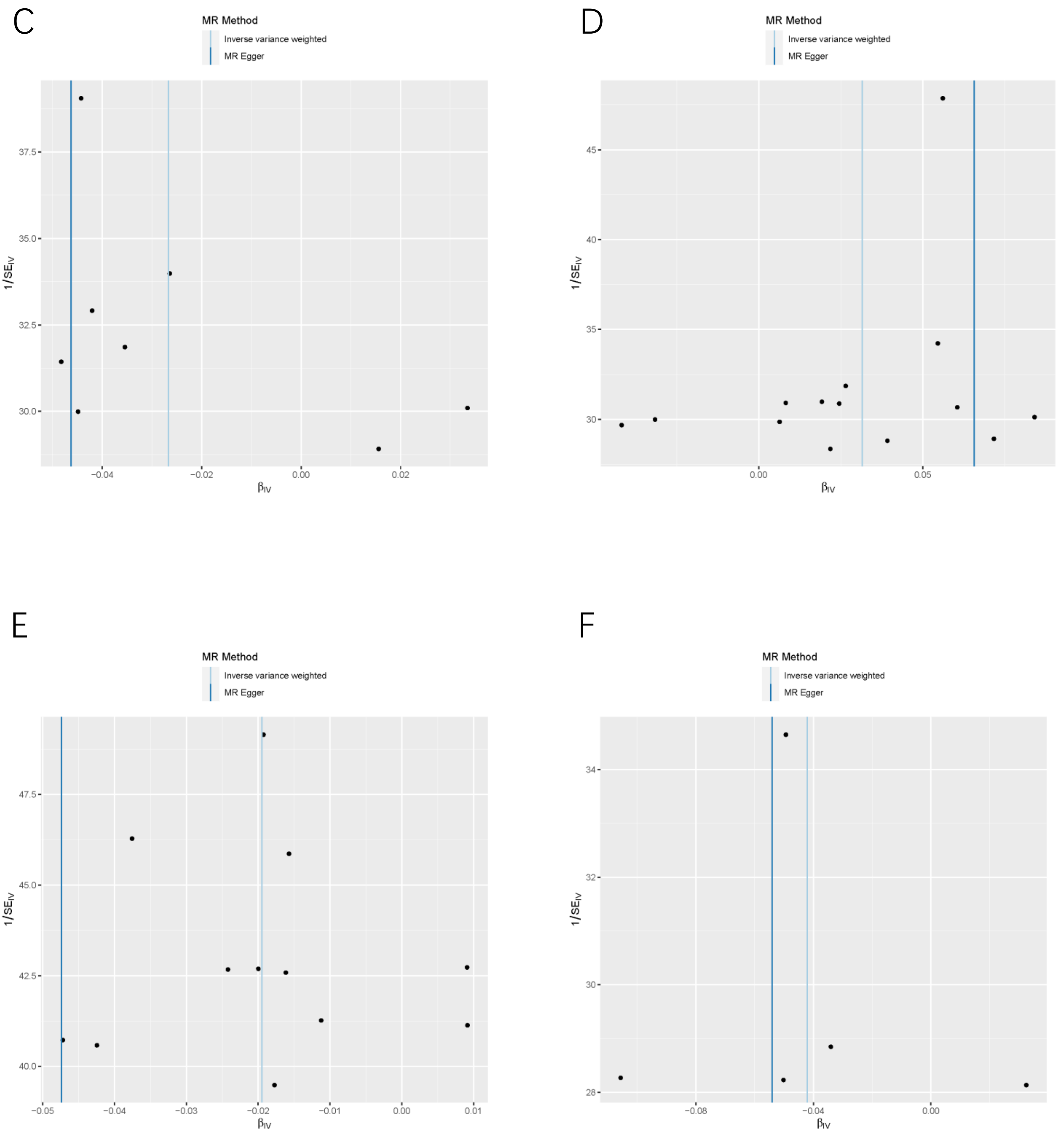
C
MR Method
Inverse variance weighted
MR Egger
1/SE$_{IV}$
β_{IV}
D
MR Method
Inverse variance weighted
MR Egger
1/SE$_{IV}$
β_{IV}
E
MR Method
Inverse variance weighted
MR Egger
1/SE$_{IV}$
β_{IV}
F
MR Method
Inverse variance weighted
MR Egger
1/SE$_{IV}$
β_{IV}

G

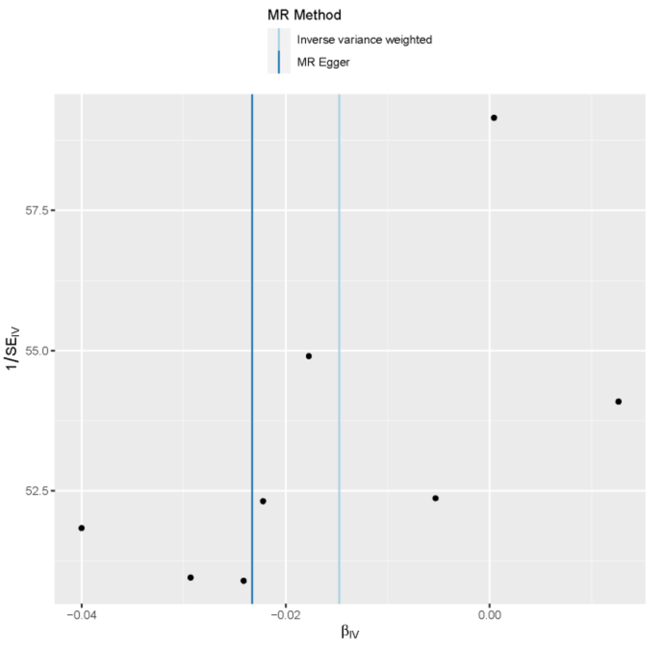

MR Method
Inverse variance weighted
MR Egger
57.5
55.0
52.5
1/SE_IV
−0.04
−0.02
0.00
β_IV

H

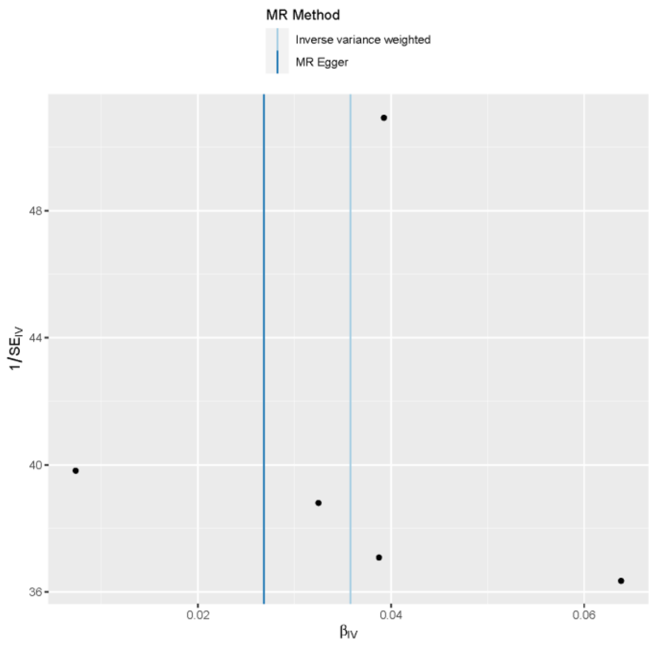

MR Method
Inverse variance weighted
MR Egger
48
44
40
36
1/SE_IV
0.02
0.04
0.06
β_IV

I

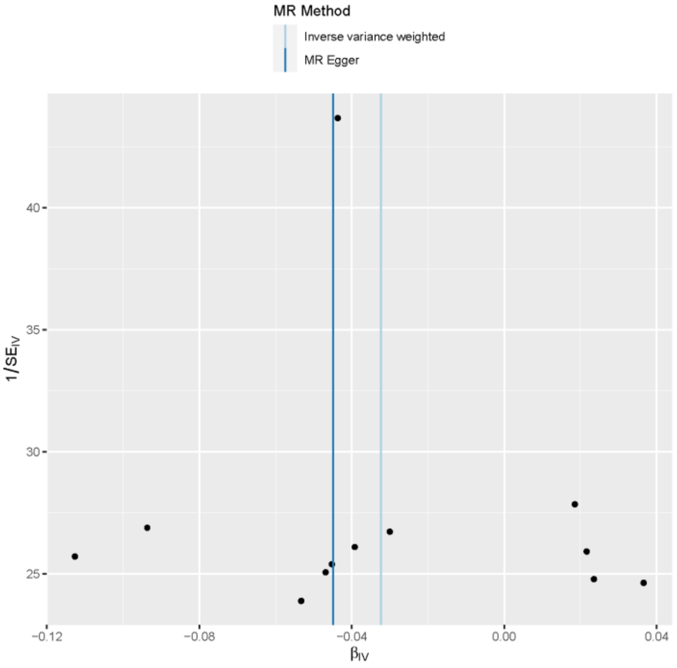

MR Method
Inverse variance weighted
MR Egger
40
35
30
25
1/SE_IV
−0.12
−0.08
−0.04
0.00
0.04
β_IV

Supplement FigureS9
Scatter plots of nominal significant estimates from genetically predicted microbiotas {
A:genus Oscillospira;
B:genus Parasutterella;
C:genus Terrisporobacter } on LDL-C

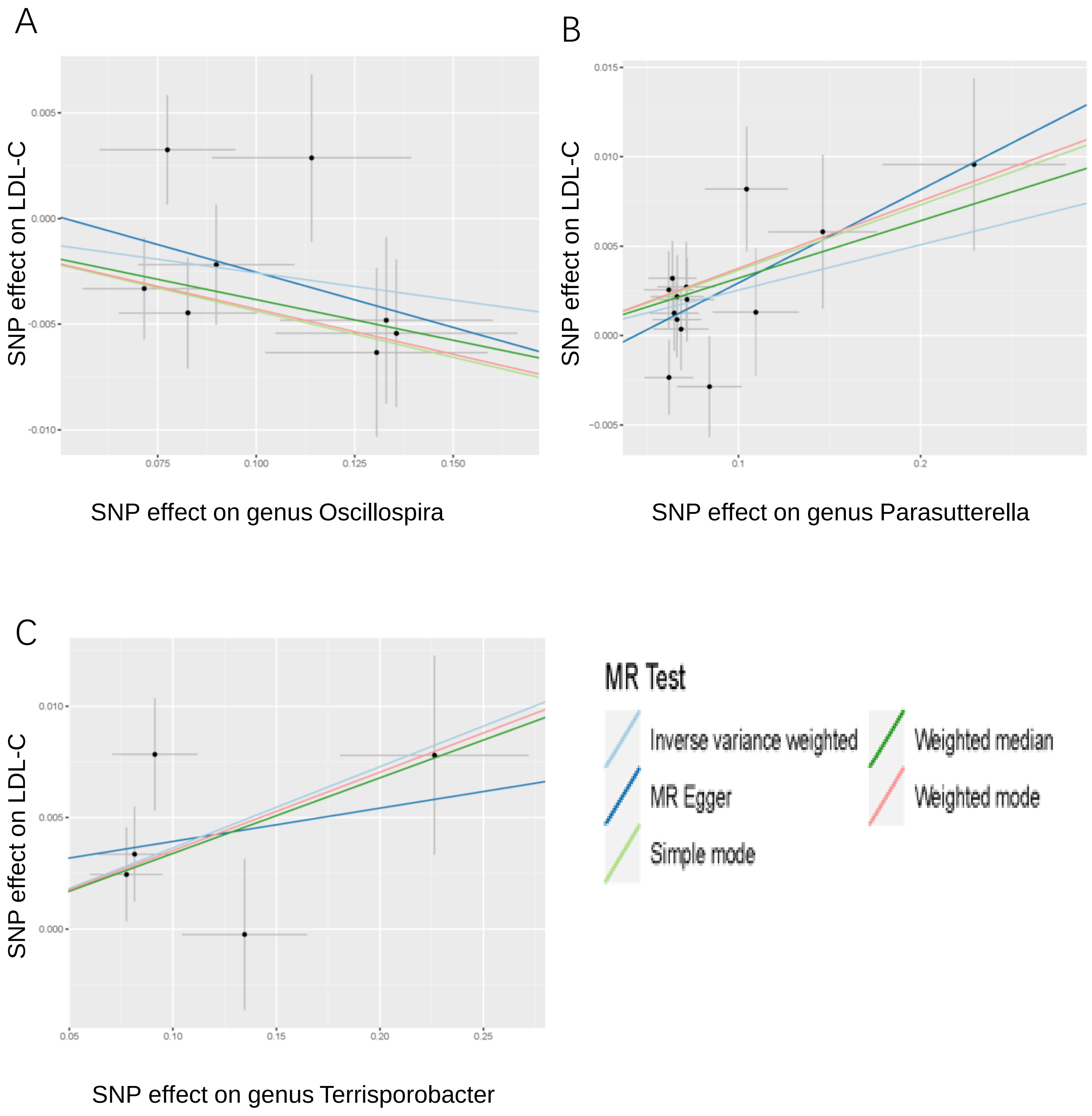

A

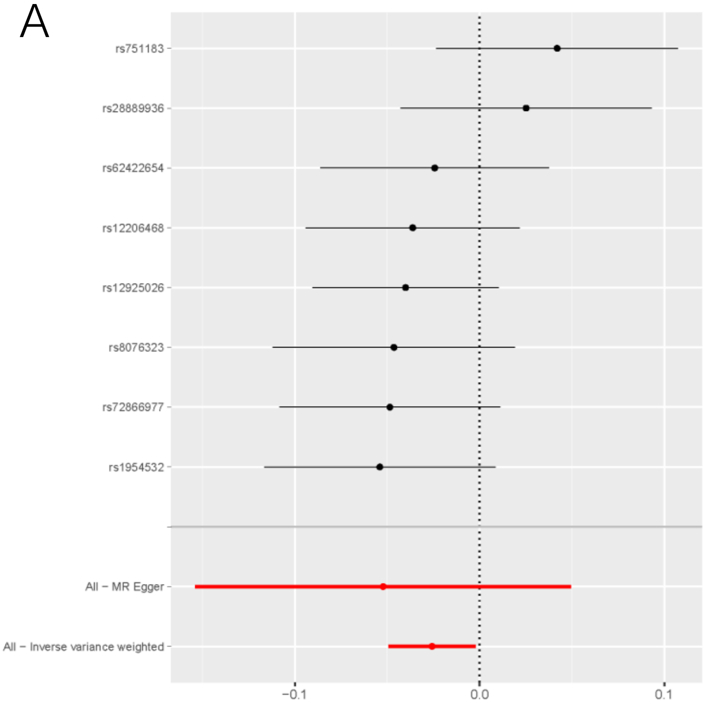

MR effect size for ‘genus Oscillospia’ on ‘LDL-C ’

B

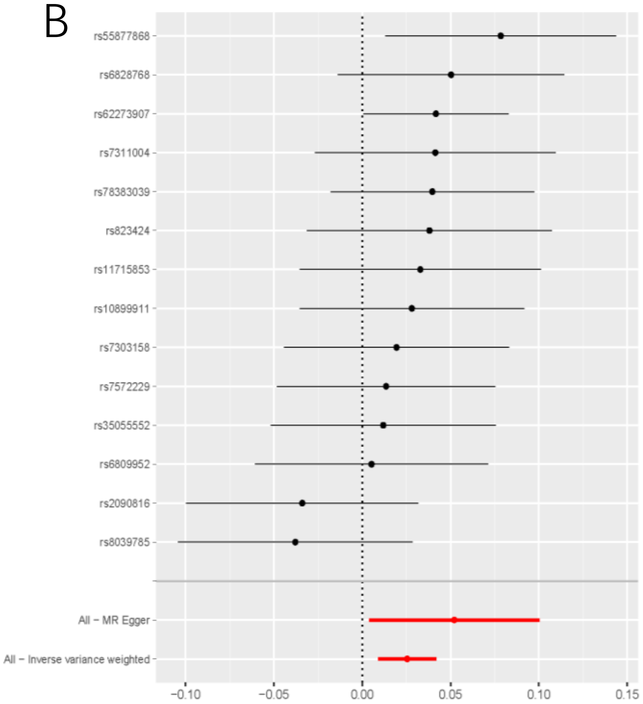

MR effect size for ‘genus Parasutterella’ on ‘LDL-C ’

C

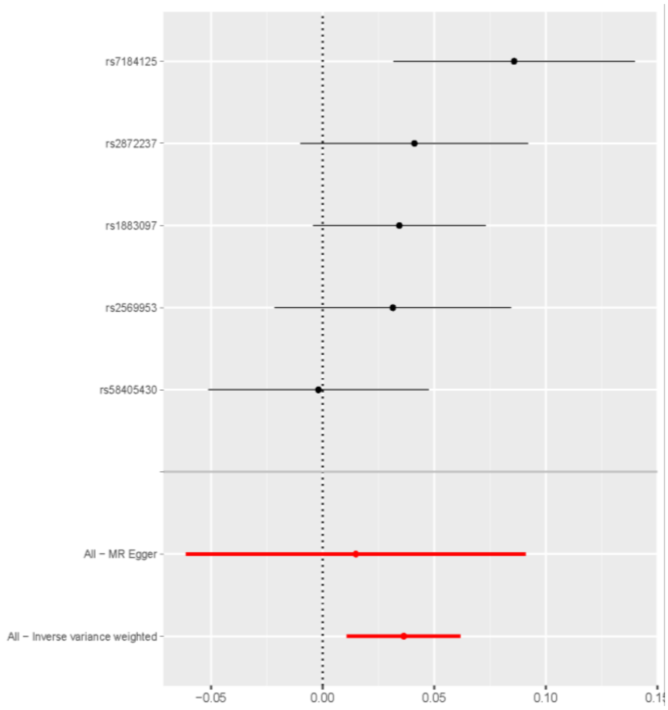

MR effect size for ‘genus Terrisporobacter ’ on ‘LDL-C ’

Supplement FigureS10 Forest plots of nominal significant estimates from genetically predicted microbiotas{
A:genus Oscillospira;
B:genus Parasutterella;
C:genus Terrisporobacter}
on LDL-C

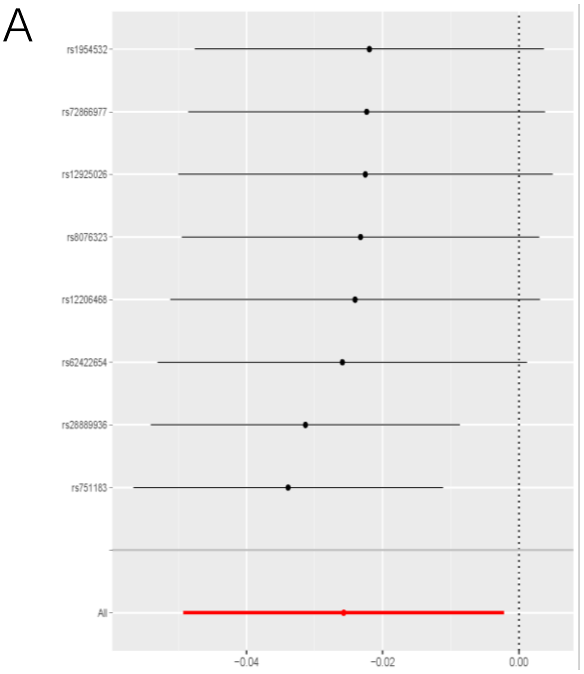

MR Leave-one-out sensitivity for ‘genus Oscillospira’ on ‘LDL-C’

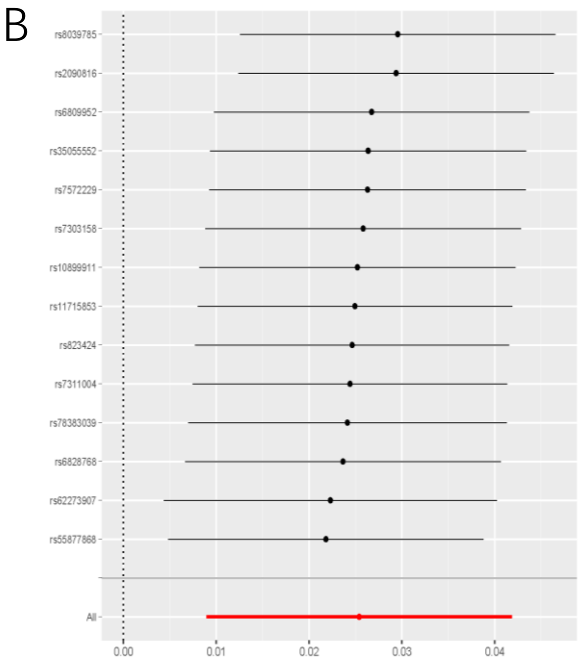

MR Leave-one-out sensitivity for ‘genus Parasutterella’ on ‘LDL-C’

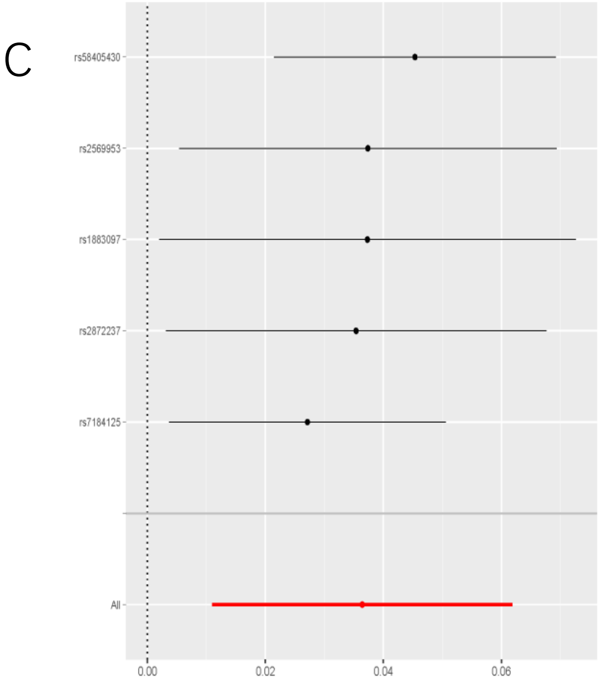

MR Leave-one-out sensitivity for ’genus Terrisporobacter’ on ‘LDL-C’

Supplement FigureS11 Leave-one-out plots of nominal significant estimates from genetically predicted microbiotas{ A:genus Oscillospira; B:genus Parasutterella; C:genus Terrisporobacter } on LDL-C

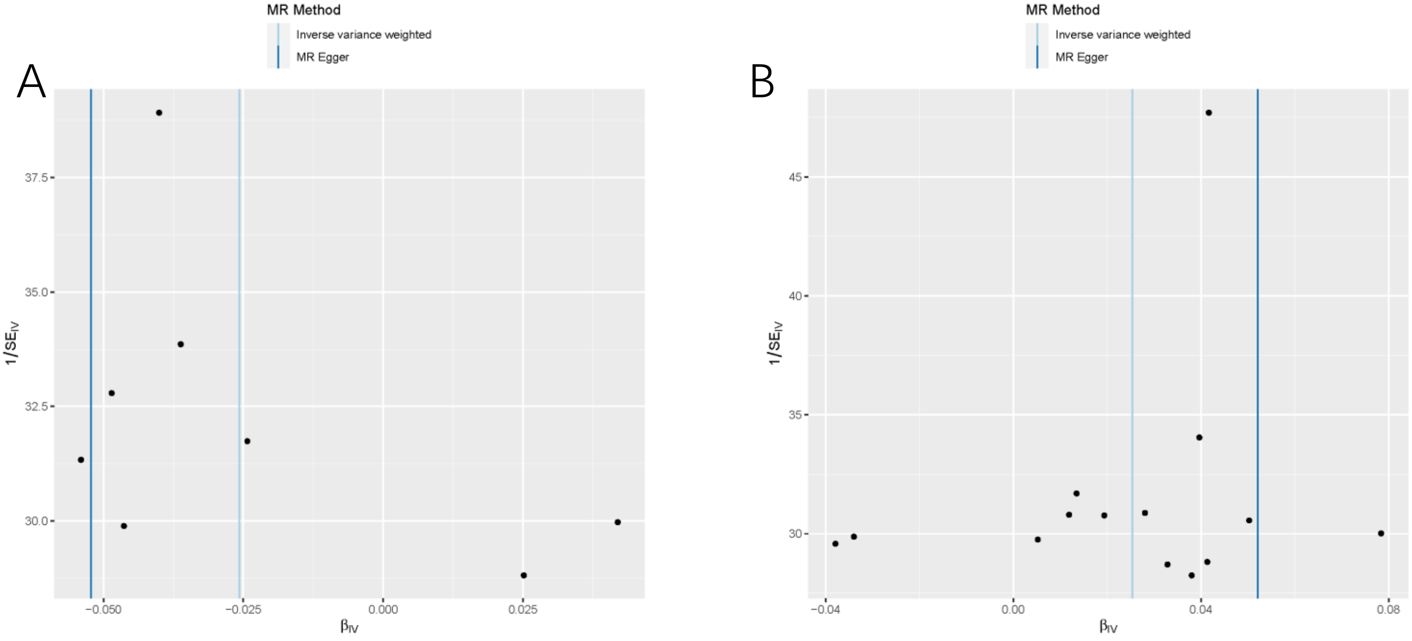

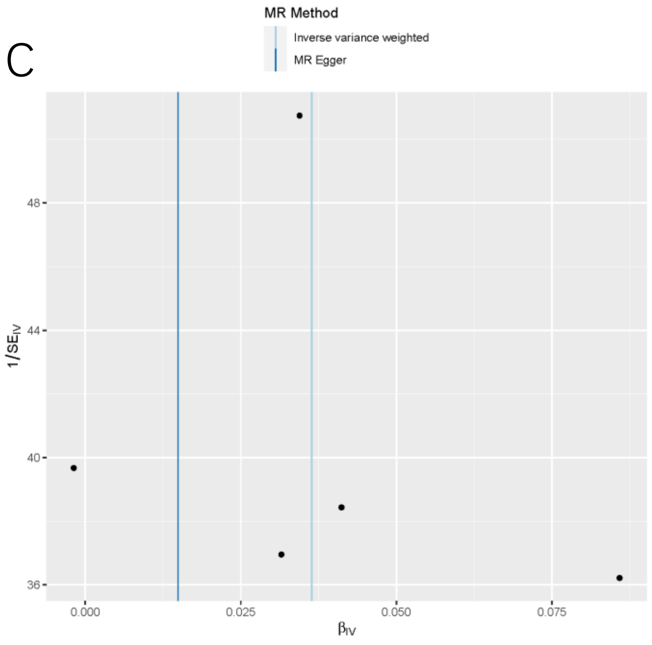

Supplement FigureS12 Funnel plots of nominal significant estimates from genetically predicted microbiotas{
A:genus Oscillospira;
B:genus Parasutterella;
C:genus Terrisporobacter}
on LDL-C

Supplement FigureS13
Scatter plots of nominal significant estimates from genetically predicted microbiotas{
A:class Erysipelotrichia;
B: family Erysipelotrichaceae;
C:genus Parabacteroides ;
D:genus Ruminococcaceae ;
E:order Erysipelotrichales}   on HDL-C

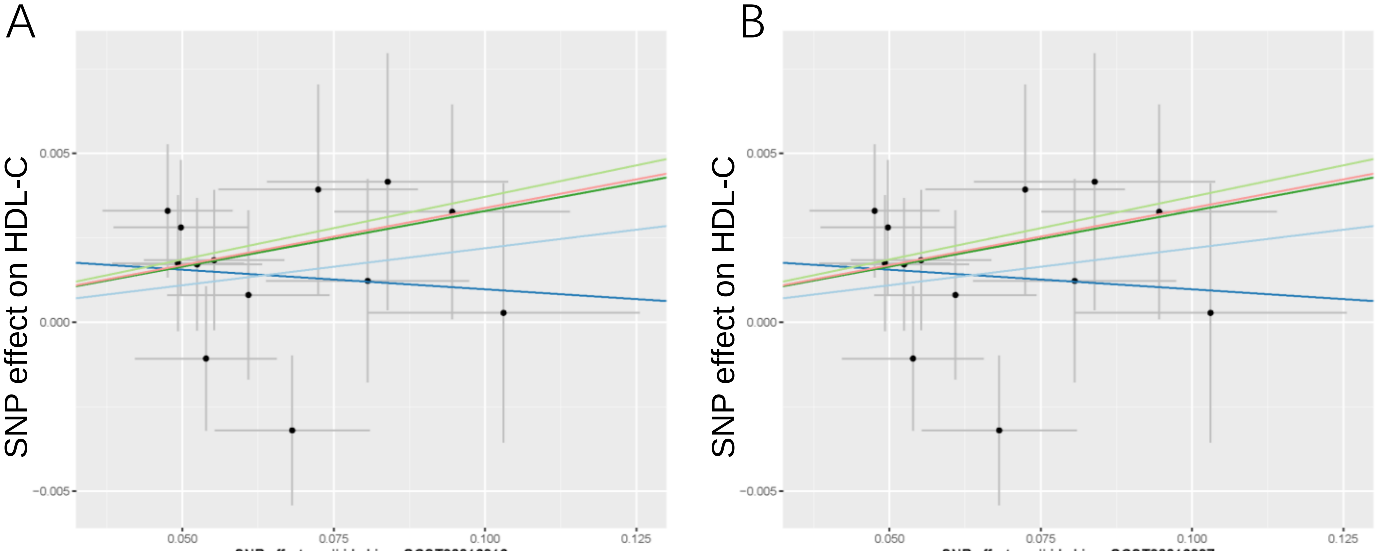

SNP effect on class Erysipelotrichia   SNP effect on family Erysipelotrichaceae

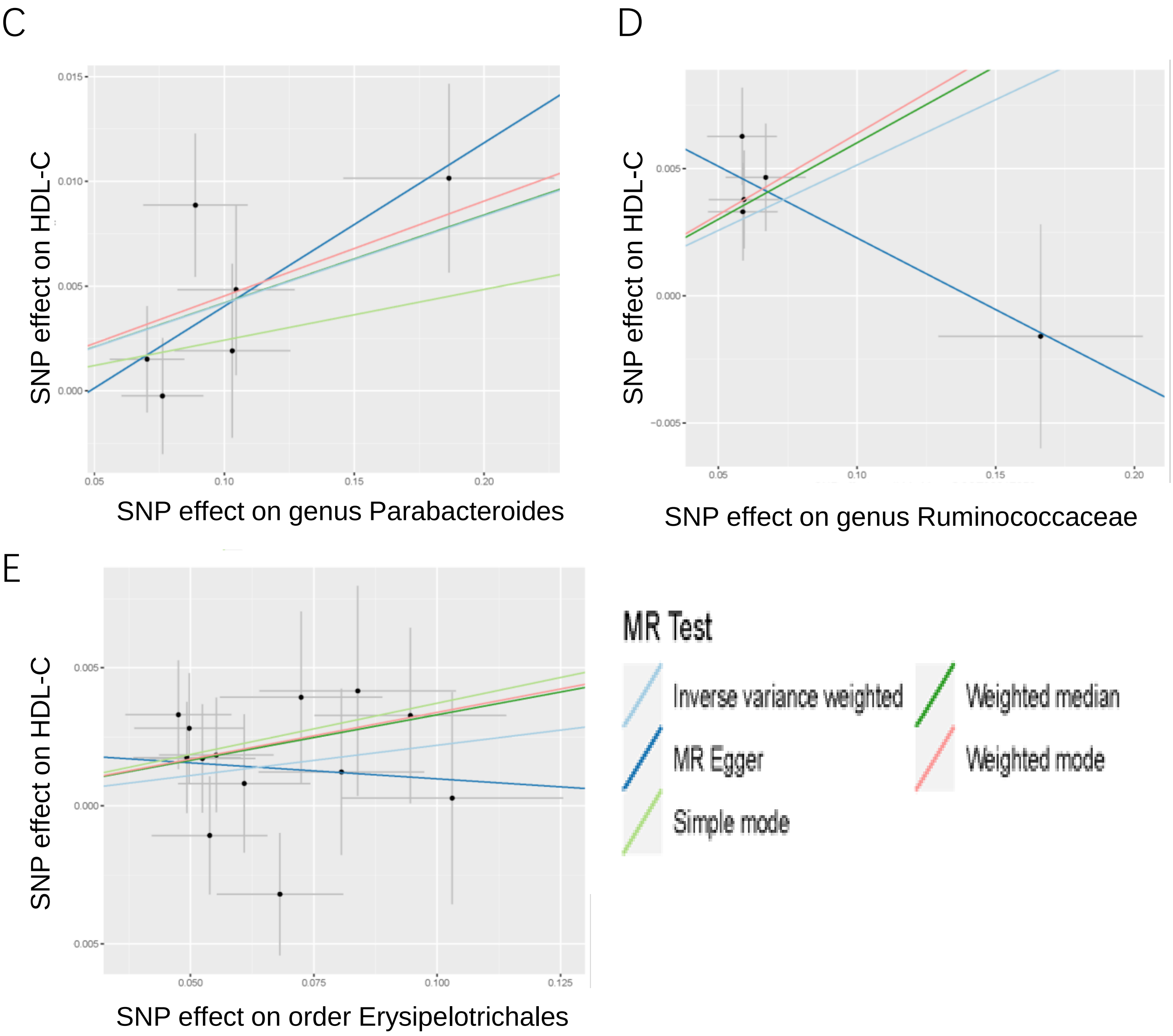
C
0.015
0.010
0.005
0.000
0.05
0.10
0.15
0.20
SNP effect on HD
SNP effect on genus Parabacteroides
D
0.005
0.000
−0.005
0.05
0.10
0.15
0.20
SNP effect on HDL-C
SNP effect on genus Ruminococcaceae
E
0.005
0.000
0.005
0.050
0.075
0.100
0.125
SNP effect on HDL-C
SNP effect on order Erysipelotrichales
MR Test
Inverse variance weighted
MR Egger
Simple mode
Weighted median
Weighted mode

Supplement FigureS14

Forest plots of nominal significant estimates from genetically predicted microbiotas{
A:class Erysipelotrichia;
B:family Erysipelotrichaceae;
C:genus Parabacteroides;
D:genus Ruminococcaceae;
E:order Erysipelotrichales} on HDL-C

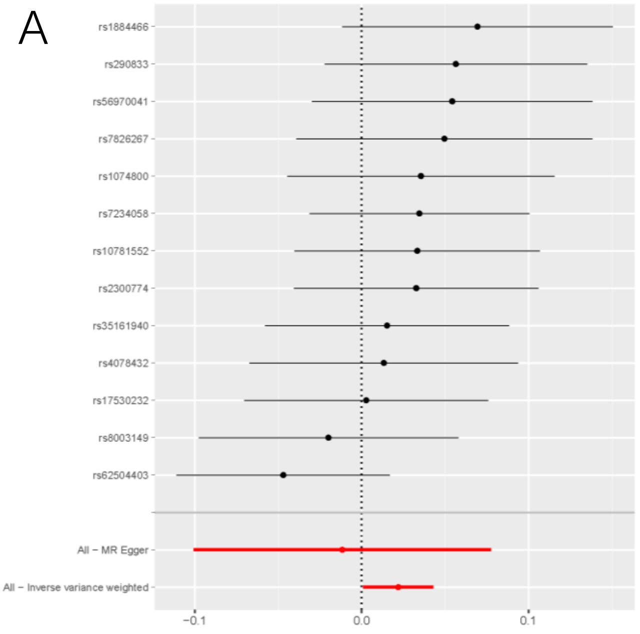

MR effect size for ‘class Erysipelotrichia’ on ‘HDL-C’

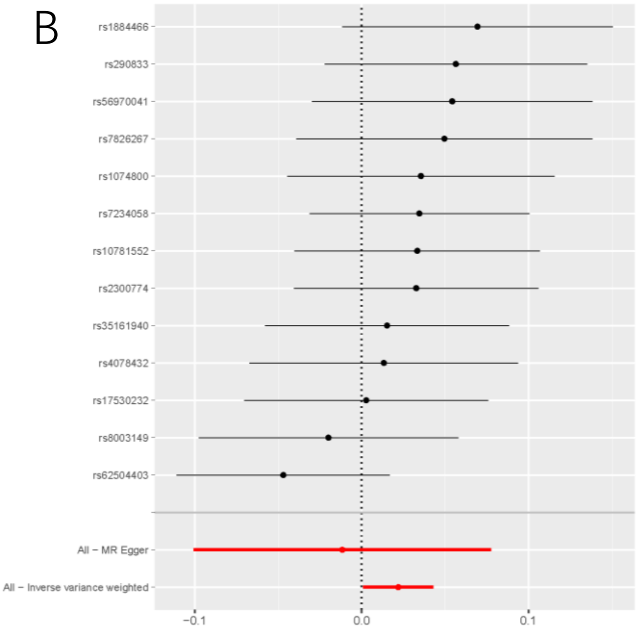

MR effect size for ‘family Erysipelotrichaceae’ on ‘HDL-C’

C

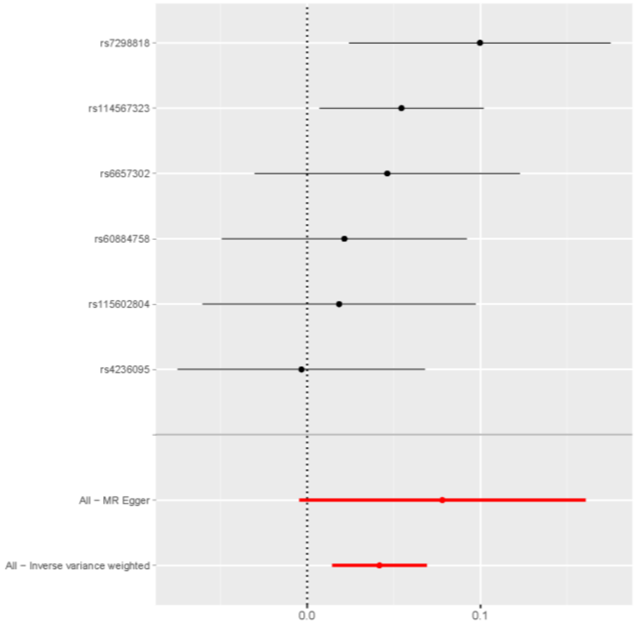

MR effect size for ‘genus Parabacteroides’ on ‘HDL-C’

D

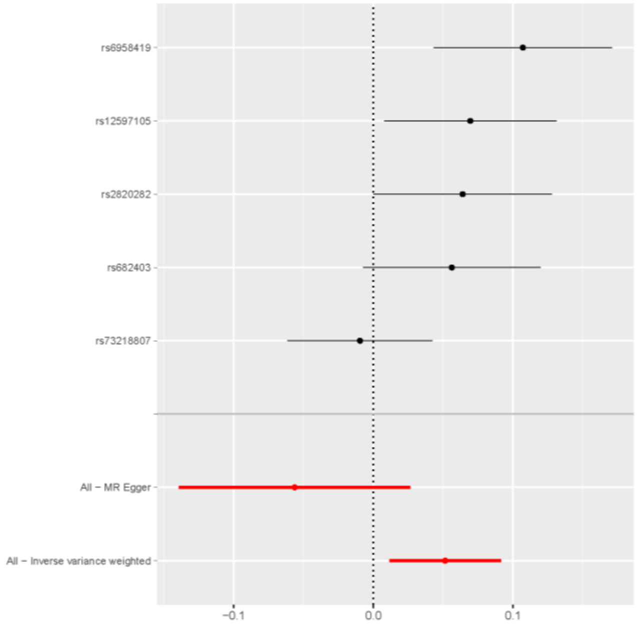

MR effect size for ‘genus Ruminococcaceae’ on ‘HDL-C’

E

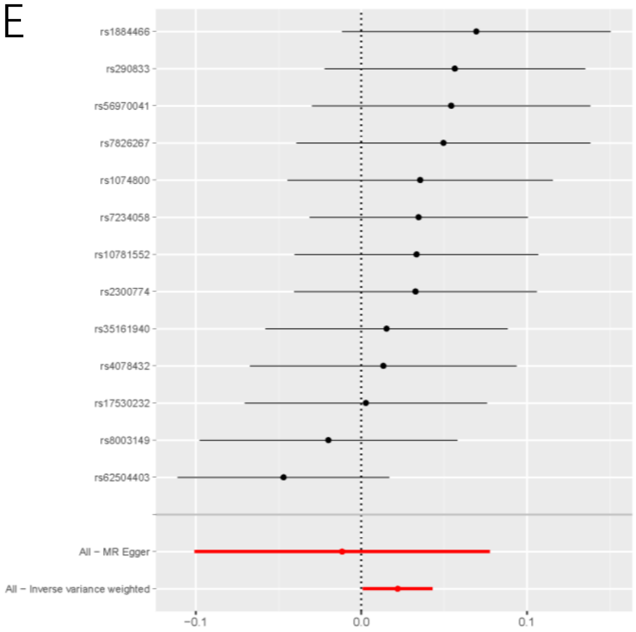

MR effect size for ‘order Erysipelotrichales’ on ‘HDL-C’

Supplement FigureS15
Leave-one-out plots of nominal significant estimates from genetically predicted microbiotas{
A:class Erysipelotrichia;
B: family Erysipelotrichaceae;
C:genus Parabacteroides ;
D:genus Ruminococcaceae;
E:order Erysipelotrichales}on HDL-C

A

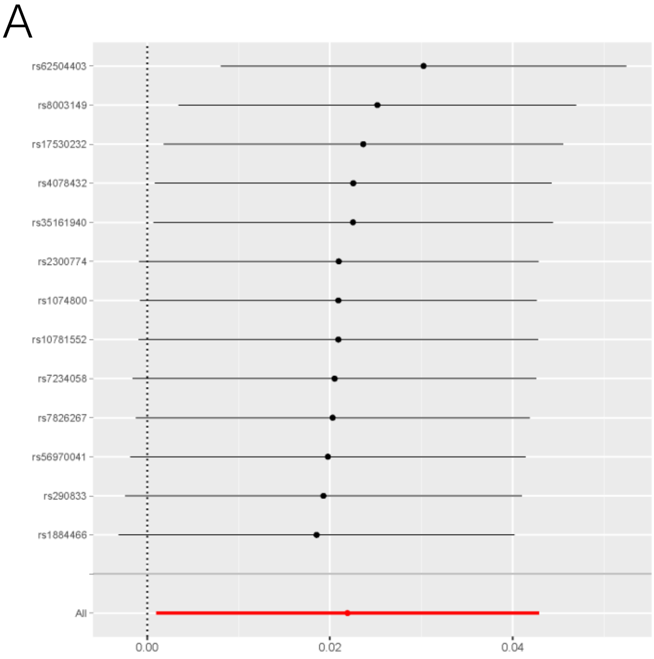

MR Leave-one-out sensitivity for ‘class Erysipelotrichia’ on ‘HDL-C’

B

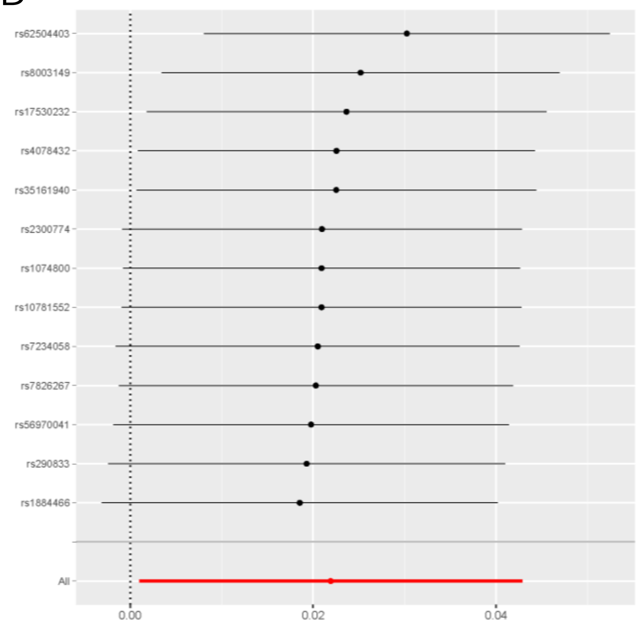

MR Leave-one-out sensitivity for ‘family Erysipelotrichaceae’ on ‘HDL-C’

C

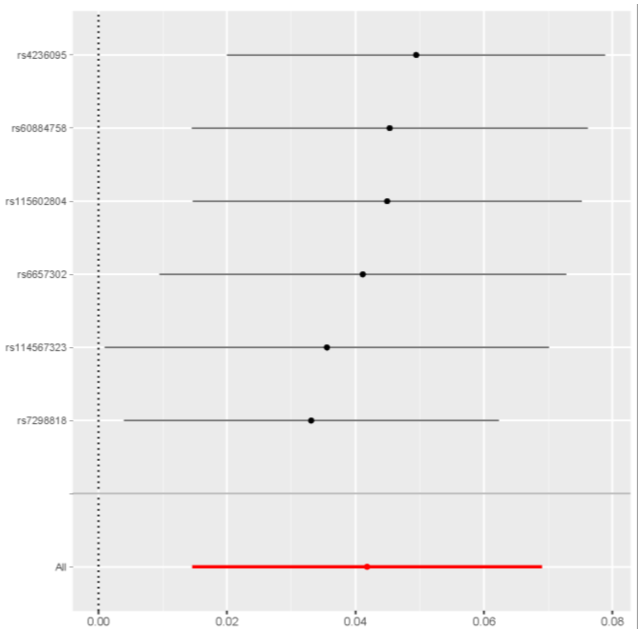

MR Leave-one-out sensitivity for ‘genus Parabacteroides ’ on ‘HDL-C’

D

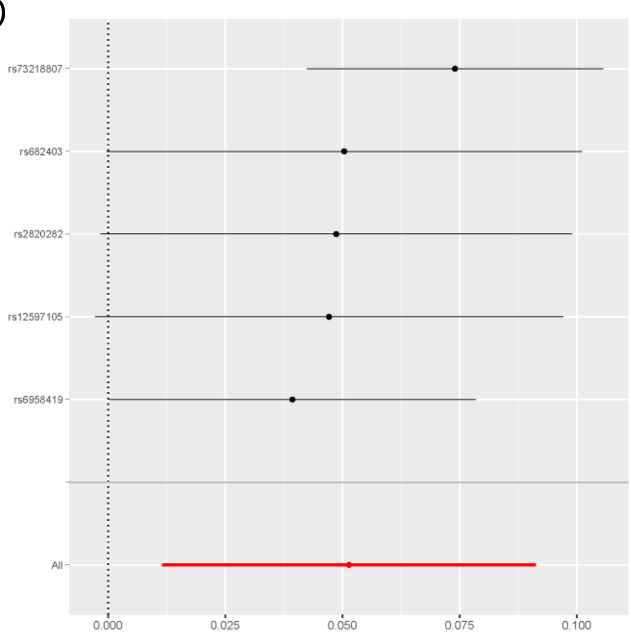

MR Leave-one-out sensitivity for ‘genus Ruminococcaceae’ on ‘HDL-C’

E

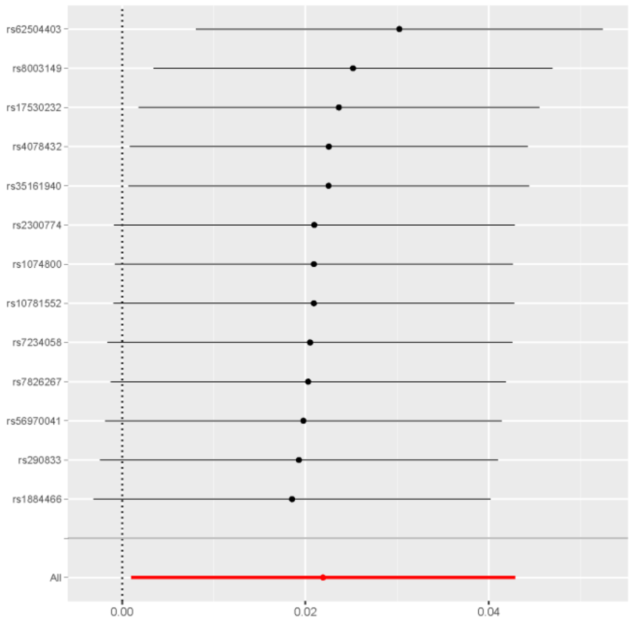

MR Leave-one-out sensitivity for ‘order Erysipelotrichales’ on ‘HDL-C’

Supplement FigureS16
 Funnel plots of nominal significant estimates from genetically predicted microbiotas{
A:class Erysipelotrichia;
B:familyErysipelotrichaceae;
C:genusParabacteroides;
D:genusRuminococcaceae;
E:order Erysipelotrichales} on HDL-C

A

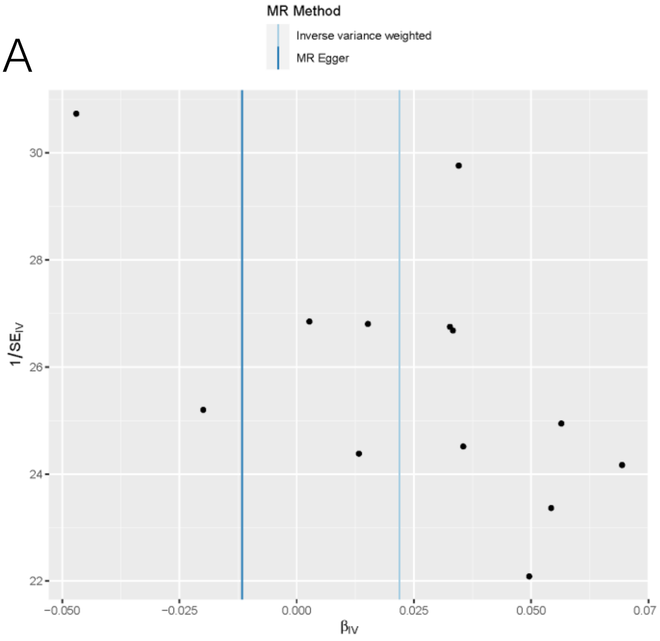

B

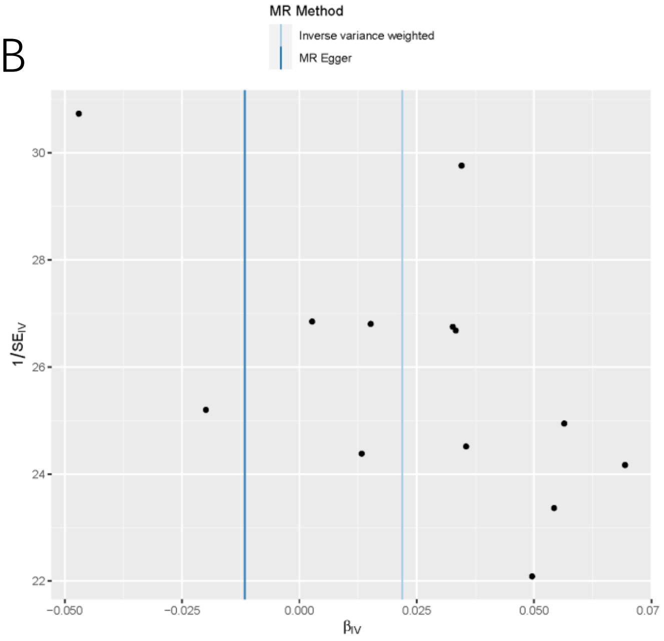

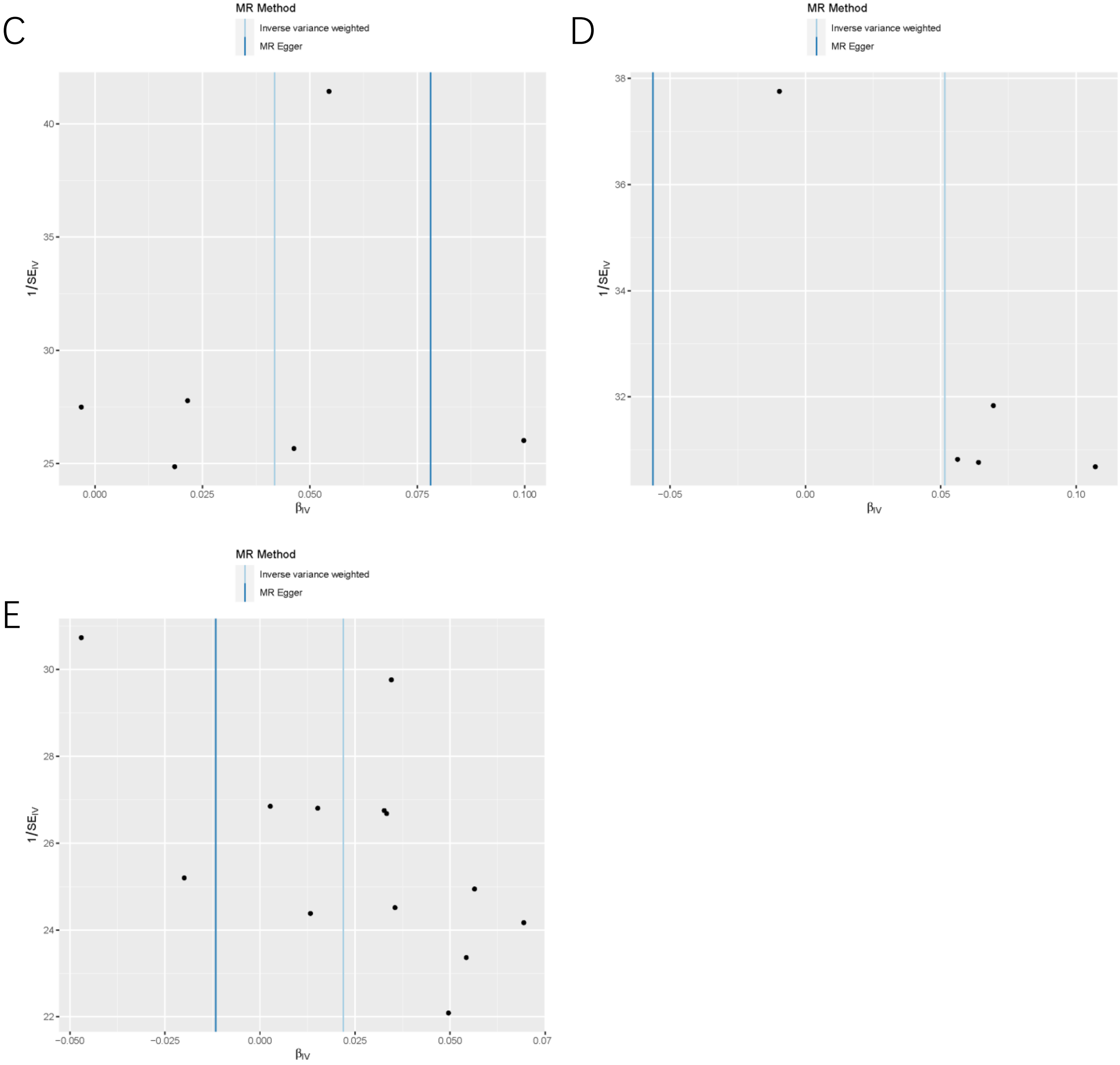

C
D
E
MR Method
Inverse variance weighted
MR Egger
1/SE_IV
β_IV

Supplement FigureS17
Scatter plots of nominal significant estimates from genetically predicted microbiotas{genus Dorea }on TG

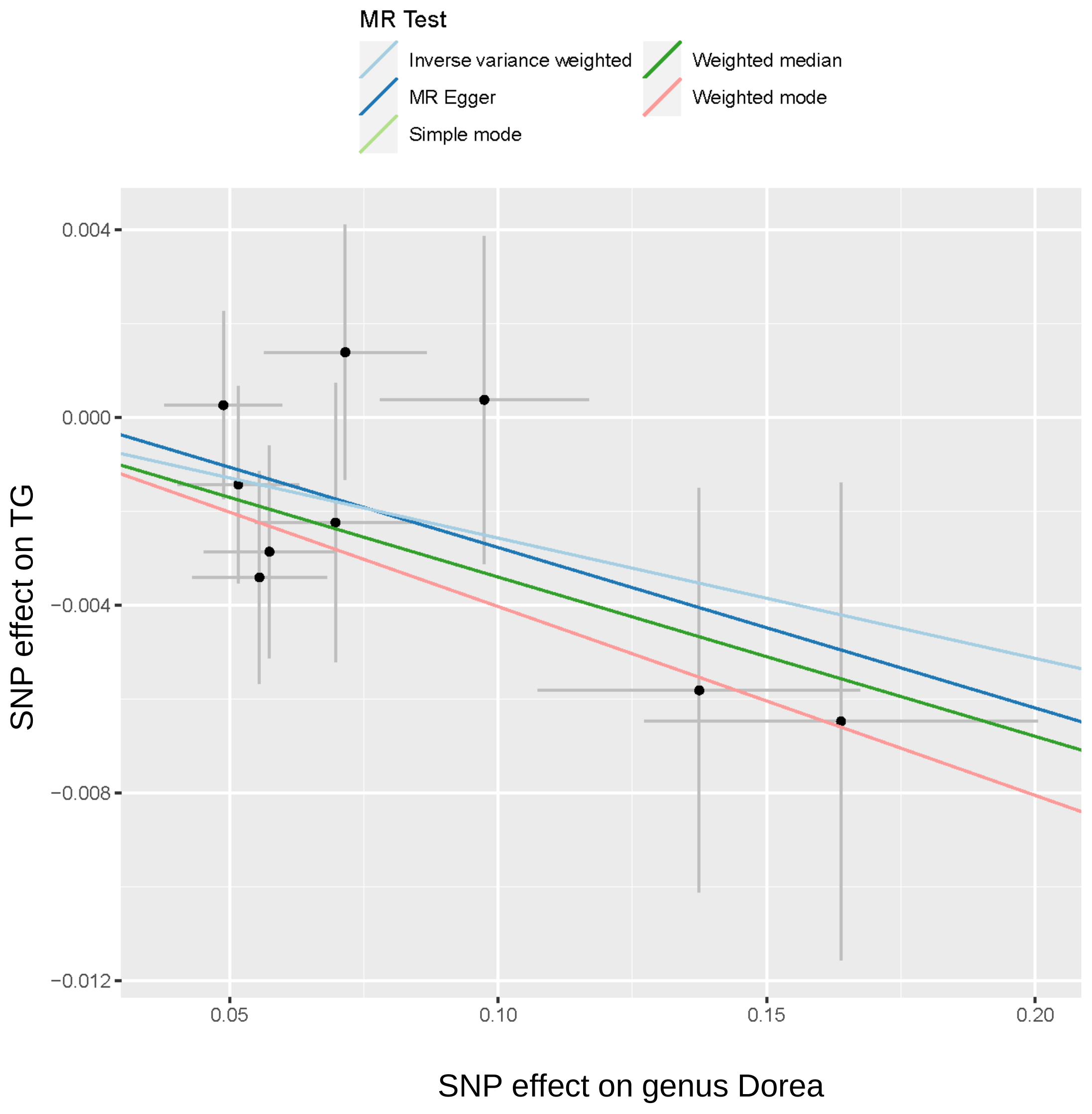

Supplement FigureS18
Forest plots of nominal significant estimates from genetically predicted microbiotas {genus Dorea } on TG

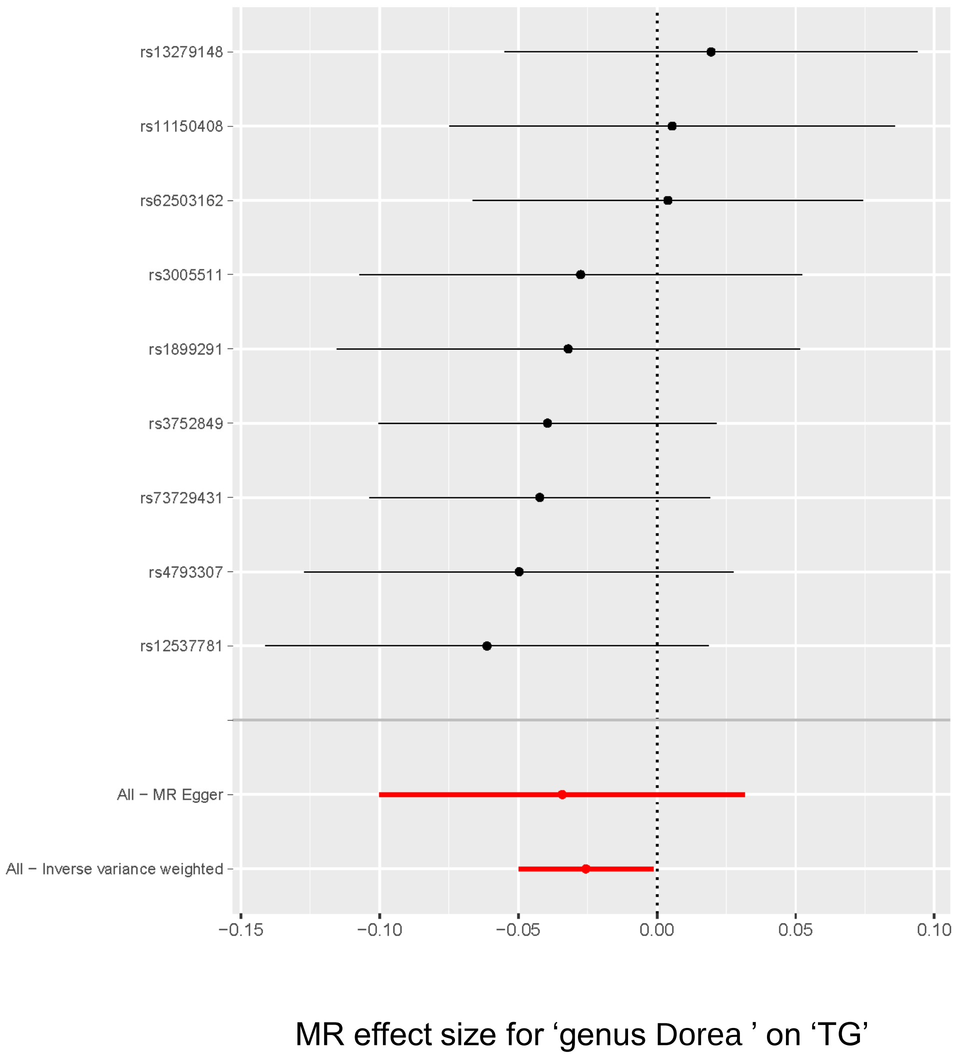

Supplement FigureS19
Leave-one-out plots of nominal significant estimates from genetically predicted microbiotas {genus Dorea } on TG

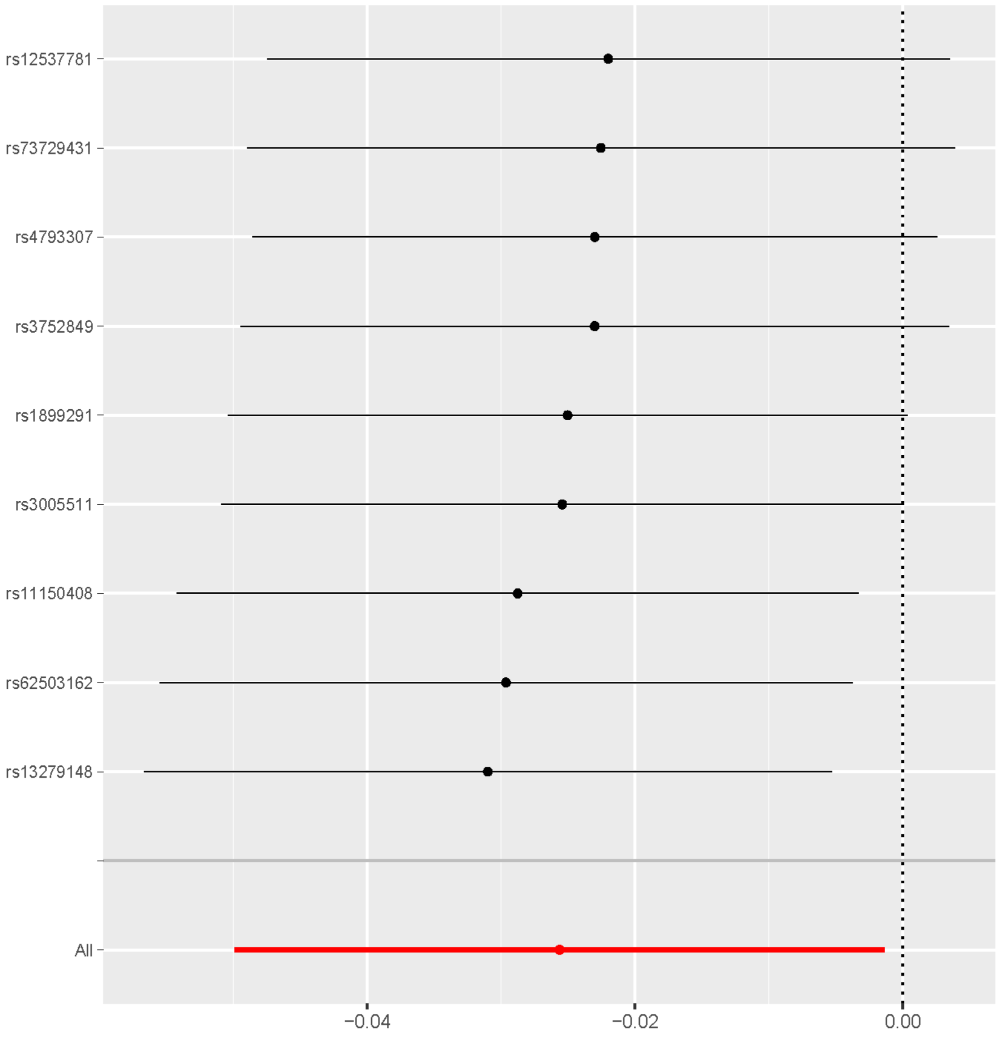

MR Leave-one-out sensitivity for ‘genus Dorea ’ on ‘TG’

Supplement FigureS20
Funnel plots of nominal significant estimates from genetically predicted microbiotas {genus Dorea } on TG

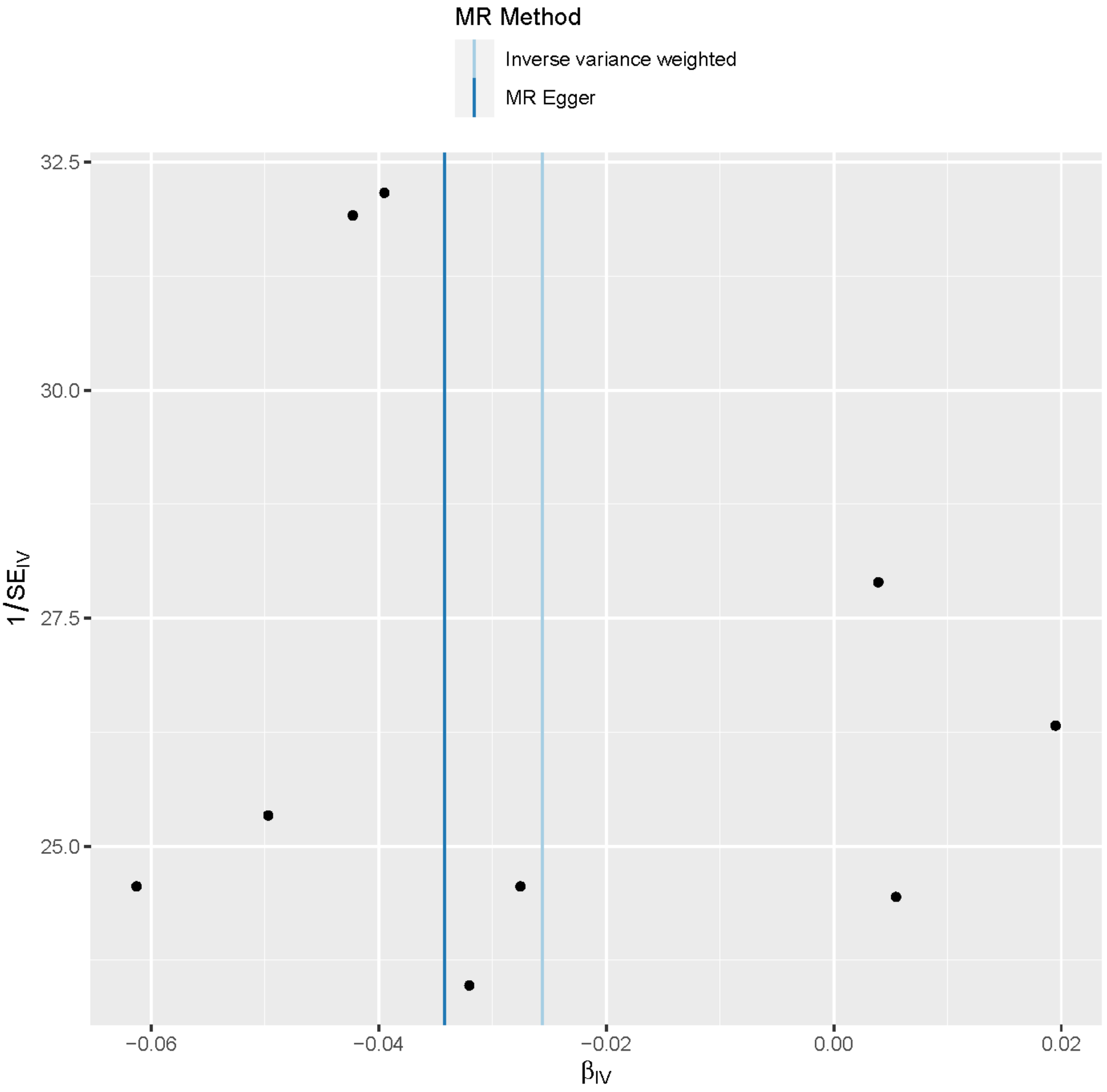

Supplement: Supplementary file 1 — Supplementary Material 1. [file 12872_2024_3804_MOESM1_ESM.zip › Supplement Figures.pdf]
