# Supplementary material for: Causality of the gut microbiome and atherosclerosis-related lipids: a bidirectional Mendelian Randomization study
Source: BMC Cardiovasc Disord. 2024 Mar 2;24:138. doi: 10.1186/s12872-024-03804-3 (PMC10909291; doi:10.1186/s12872-024-03804-3)
Supplement: Supplementary file 1 — Supplementary Material 1. [file 12872_2024_3804_MOESM1_ESM.zip › STROBE-MR .docx]

**STROBE-MR checklist of recommended items to address in reports of Mendelian randomization studies**^1^ ^2^

| **Item No.** | **Section** | **Checklist item** | **Page No.** | **Relevant text from manuscript** |
| --- | --- | --- | --- | --- |
| 1 | **TITLE and ABSTRACT** | Indicate Mendelian randomization (MR) as the study’s design in the title and/or the abstract if that is a main purpose of the study | 1,2 | Title：  Causality of the gut microbiome and atherosclerosis-related lipids: A bidirectional Mendelian randomization study  Abstract：  Aims: Some observational and clinical studies have indicated an association between intestinal flora and lipids. However, the causal relationship between intestinal flora and blood lipids remains unclear. To investigate the causal relationships between the gut microbiome and intestinal flora and blood lipids by using genetics.  Methods: We performed a bidirectional two-sample Mendelian Randomization (MR) analysis to investigate the causal relationship between intestinal flora and blood lipids. Summary statistics of genome-wide association studies (GWASs) for the 211 intestinal flora and blood lipid traits were obtained from public datasets. Single-nucleotide polymorphisms (SNPs) significantly associated with intestinal flora were selected as exposure instrumental variables (IVs). Five recognized MR methods were applied to assess the causal relationship with lipids, among which, the inverse-variance weighted (IVW) regression was used as the primary MR method. A series of sensitivity analyses were performed to test the robustness of the causal estimates.  Results: The results indicated a potential causal association between 19 intestinal flora and dyslipidemia in humans. Genus Ruminococcaceae, Christensenellaceae, Parasutterella, Terrisporobacter, Parabacteroides, Class Erysipelotrichia, Family Erysipelotrichaceae, and order Erysipelotrichales were associated with higher lipids, whereas genus Oscillospira, Peptococcus, Ruminococcaceae UCG010, Ruminococcaceae UCG011, Dorea, and Family Desulfovibrionaceae were associated with lower lipids. After using the Bonferroni method for multiple testing correction, Only Desulfovibrionaceae [Estimate = -0.0418, 95% confidence interval [CI]: 0.9362-0.9826, P = 0.0007] exhibited stable and significant negative associations with ApoB levels. The inverse MR analysis did not find a significant causal effect of lipids on the intestinal flora. Additionally, no significant heterogeneity or horizontal pleiotropy for IVs was observed in the analysis.  Conclusion: The study suggested a causal relationship between intestinal flora and dyslipidemia. These findings will provide a meaningful reference to discover dyslipidemia for intervention to address the problems in the clinic. |
|  | **INTRODUCTION** |  |  |  |
| 2 | **Background** | Explain the scientific background and rationale for the reported study. What is the exposure? Is a potential causal relationship between exposure and outcome plausible? Justify why MR is a helpful method to address the study question | 2 | The intestinal flora consists of approximately 4 × 10^13^ commensal bacteria, also known as the “human second genome”. Studies have pointed that intestinal flora has a closely relationship with lipid levels. Mendelian randomization (MR) analysis, which employs genetic variation as instrumental variables (IVs), is a method for inferring causal relationships between exposure and outcome. MR analysis can overcome the effects of confounding factors, such as behavioral and environmental factors. Moreover, it can provide reliable evidence for causal relationships between risk factors and diseases. |
| 3 | **Objectives** | State specific objectives clearly, including pre-specified causal hypotheses (if any). State that MR is a method that, under specific assumptions, intends to estimate causal effects |  | This study employs Two-sample MR to assess causal relationships between intestinal flora and lipids, utilizing the latest genome-wide association study (GWAS) data that covers a wide range of populations, and evaluating the reliability of MR results. |
|  | **METHODS** |  |  |  |
| 4 | **Study design and data sources** | Present key elements of the study design early in the article. Consider including a table listing sources of data for all phases of the study. For each data source contributing to the analysis, describe the following: | 3 | This is an MR study investigating the causal relationship between between intestinal flora and lipids. The MR study relies on the strict adherence to three assumptions: (1) the relevance assumption, where IVs should be strongly associated with the exposure; (2) the independence assumption, where the effect of IVs on the outcome can only be mediated through the exposure; (3) the exclusion restriction assumption, where IVs should not have a direct association with the outcome. Moreover, we have followed the recommendations of Strengthening the Reporting of Observational Studies in Epidemiology Using Mendelian Randomization (STROBE-MR) to ensure the replicability of our study.  The R package TwoSampleMR was utilized for conducting the MR analysis, while the R package MR-PRESSO was employed for performing MR-PRESSO. All the aforementioned analyses were carried out on R software version 4.1.2 |
|  | a) | Setting: Describe the study design and the underlying population, if possible. Describe the setting, locations, and relevant dates, including periods of recruitment, exposure, follow-up, and data collection, when available. | 3 | Our exposure sample was obtained from the UK Biobank (UKBB) project (<https://www.ukbiobank.ac.uk/>), in which 18340 participants from 24cohorts  For the outcome dataset, GWAS data for lipids were obtained from IEU Open GWAS Database([IEU OpenGWAS project (mercies. ac. UK)](https://gwas.mrcieu.ac.uk/)) are included 441,016 participants  More summary statistics about the exposure and outcome are presented in the Supplementary table S1-S2. |
|  | b) | Participants: Give the eligibility criteria, and the sources and methods of selection of participants. Report the sample size, and whether any power or sample size calculations were carried out prior to the main analysis | 3 | Our exposure sample was obtained from the UK Biobank (UKBB) project (<https://www.ukbiobank.ac.uk/>), in which 18340 participants from 24cohorts  For the outcome dataset, GWAS data for lipids were obtained from IEU Open GWAS Database([IEU OpenGWAS project (mercies. ac. UK)](https://gwas.mrcieu.ac.uk/)) are included 441,016 participants  More summary statistics about the exposure and outcome are presented in the Supplementary table S1-S2 |
|  | c) | Describe measurement, quality control and selection of genetic variants | 4 | We selected SNPs with a genome-wide association (p< 1E-05), with independent inheritance (r^2^< 0.01), and without linkage disequilibrium (LD) in summary statistics. We also calculated the F statistic for each exposure to avoid bias due to weak genetic instruments. IVs with an F statistic of less than 10 were excluded and were often labeled as “weak instruments”. Moreover, the outliers of missing data were excluded. |
|  | d) | For each exposure, outcome, and other relevant variables, describe methods of assessment and diagnostic criteria for diseases | / | / |
|  | e) | Provide details of ethics committee approval and participant informed consent, if relevant | / | / |
| 5 | **Assumptions** | Explicitly state the three core IV assumptions for the main analysis (relevance, independence and exclusion restriction) as well assumptions for any additional or sensitivity analysis | 3,4 | The MR study relies on the strict adherence to three assumptions: (1) the relevance assumption, where IVs should be strongly associated with the exposure; (2) the independence assumption, where the effect of IVs on the outcome can only be mediated through the exposure; (3) the exclusion restriction assumption, where IVs should not have a direct association with the outcome.  We conducted the MR-Egger regression to evaluate the possibility of horizontal pleiotropy. The intercept term of the MR-Egger regression shows the mean pleiotropic effect IV. To assess the heterogeneity of the effects we used Cochran's Q test with IVW and MR-Egger, and P< 0.05 determined by Cochran's Q test was considered heterogeneous. The Leave-one-out sensitivity analysis was performed to test the robustness of the association results by removing studies individually. |
| 6 | **Statistical methods: main analysis** | Describe statistical methods and statistics used |  |  |
|  | a) | Describe how quantitative variables were handled in the analyses (i.e., scale, units, model) | / | / |
|  | b) | Describe how genetic variants were handled in the analyses and, if applicable, how their weights were selected | 3,4 | We selected SNPs with a genome-wide association (p< 1E-05), with independent inheritance (r2< 0.01), and without linkage disequilibrium (LD) in summary statistics. We also calculated the F statistic for each exposure to avoid bias due to weak genetic instruments. IVs with an F statistic of less than 10 were excluded and were often labelled as “weak instruments” Moreover, the outliers of missing data were excluded. |
|  | c) | Describe the MR estimator (e.g. two-stage least squares, Wald ratio) and related statistics. Detail the included covariates and, in case of two-sample MR, whether the same covariate set was used for adjustment in the two samples | 4 | After identifying the genetic instruments for each exposure, genetic variants associated with intestinal flora were selected as genetic instruments for each exposure. We first applied MR-PRESSO to detect and correct for any outliers reflecting likely pleiotropic biases for all reported results. Two-sample MR analysis was performed using five different methods, including Inverse Variance Weighted (IVW), Weighted median, MR-Egger, Simple mode, and Weighted mode. Each method makes different assumptions on the validity of IVs, but the IVW method is generally considered the most reliable. IVW was used as our principal model, which accounts for heterogeneity in the variant-specific causal estimates. The other methods were used as complementary or to observe if their results were consistent with the direction of IVW. Finally, a forest plot was used for visualization. |
|  | d) | Explain how missing data were addressed | 4 | Moreover, the outliers of missing data were excluded. |
|  | e) | If applicable, indicate how multiple testing was addressed | / | / |
| 7 | **Assessment of assumptions** | Describe any methods or prior knowledge used to assess the assumptions or justify their validity | 3,4 | To satisfy the three strict assumptions mentioned earlier, we performed a series of quality control steps to select suitable SNPs. We selected SNPs with a genome-wide association (p< 1E-05), with independent inheritance (r2< 0.01), and without linkage disequilibrium (LD) in summary statistics. We also calculated the F statistic for each exposure to avoid bias due to weak genetic instruments. IVs with an F statistic of less than 10 were excluded and were often labeled as “weak instruments”  We conducted the MR-Egger regression to evaluate the possibility of horizontal pleiotropy. The intercept term of the MR-Egger regression shows the mean pleiotropic effect IV. To assess the heterogeneity of the effects we used Cochran's Q test with IVW and MR-Egger, and P< 0.05 determined by Cochran's Q test was considered heterogeneous. The Leave-one-out sensitivity analysis was performed to test the robustness of the association results by removing studies individually. |
| 8 | **Sensitivity analyses and additional analyses** | Describe any sensitivity analyses or additional analyses performed (e.g. comparison of effect estimates from different approaches, independent replication, bias analytic techniques, validation of instruments, simulations) | 4 | In this study, sensitivity analyses were performed using different methods. We conducted the MR-Egger regression to evaluate the possibility of horizontal pleiotropy. The intercept term of the MR-Egger regression shows the mean pleiotropic effect IV. To assess the heterogeneity of the effects we used Cochran's Q test with IVW and MR-Egger, and P< 0.05 determined by Cochran's Q test was considered heterogeneous. The Leave-one-out sensitivity analysis was performed to test the robustness of the association results by removing studies individually. |
| 9 | **Software and pre-registration** |  |  |  |
|  | a) | Name statistical software and package(s), including version and settings used | 3 | The R package TwoSampleMR was utilized for conducting the MR analysis, while the R package MR-PRESSO was employed for performing MR-PRESSO. All the aforementioned analyses were carried out on R software version 4.2.1. |
|  | b) | State whether the study protocol and details were pre-registered (as well as when and where) | / | / |
|  | **RESULTS** |  |  |  |
| 10 | **Descriptive data** |  |  |  |
|  | a) | Report the numbers of individuals at each stage of included studies and reasons for exclusion. Consider use of a flow diagram | 3 | Our exposure sample was obtained from the UK Biobank (UKBB) project (<https://www.ukbiobank.ac.uk/>), in which 18340 participants from 24cohorts  For the outcome dataset, GWAS data for lipids were obtained from IEU Open GWAS Database([IEU OpenGWAS project (mercies. ac. UK)](https://gwas.mrcieu.ac.uk/)) are included 441,016 participants  More summary statistics about the exposure and outcome are presented in the Supplementary table S1-S2.  The flow diagram showed in Figure1 |
|  | b) | Report summary statistics for phenotypic exposure(s), outcome(s), and other relevant variables (e.g. means, SDs, proportions) | 3 | Our exposure sample was obtained from the UK Biobank (UKBB) project (<https://www.ukbiobank.ac.uk/>), in which 18340 participants from 24cohorts  For the outcome dataset, GWAS data for lipids were obtained from IEU Open GWAS Database([IEU OpenGWAS project (mercies. ac. UK)](https://gwas.mrcieu.ac.uk/)) are included 441,016 participants  More summary statistics about the exposure and outcome are presented in the Supplementary table S1-S2. |
|  | c) | If the data sources include meta-analyses of previous studies, provide the assessments of heterogeneity across these studies | / | / |
|  | d) | For two-sample MR:  i.  Provide justification of the similarity of the genetic variant-exposure associations between the exposure and outcome samples  ii.  Provide information on the number of individuals who overlap between the exposure and outcome studies | 3 | In this study, the sample for the GWAS of intestinal flora primarily comes from the UK Biobank (UKBB).The sample for the GWAS of lipids also from the IEU Open GWAS Database.But the GWAS data for exposure and outcomes were derived from two largely independent samples, making sample overlap and its impact on the study results potentially negligible.  The GWAS summary data is derived from a database of samples from European populations and there is little potential for sample overlap. The source of the data was also approved by the appropriate ethics committee, and therefore no ethical approval was required for this study |
| 11 | **Main results** |  |  |  |
|  | a) | Report the associations between genetic variant and exposure, and between genetic variant and outcome, preferably on an interpretable scale | / | / |
|  | b) | Report MR estimates of the relationship between exposure and outcome, and the measures of uncertainty from the MR analysis, on an interpretable scale, such as odds ratio or relative risk per SD difference | 5 | After identifying and removing abnormal SNPs, the results indicated a potential causal association between 19 intestinal flora and dyslipidemia in humans. Genus *Ruminococcaceae, Christensenellaceae, Parasutterella, Terrisporobacter, Parabacteroides,* Class Erysipelotrichia, Family Erysipelotrichaceae, and order Erysipelotrichales were associated with higher lipids, whereas genus *Oscillospira, Peptococcus, Ruminococcaceae UCG010, Ruminococcaceae UCG011, Dorea*, and Family Desulfovibrionaceae were associated with lower lipids. Among them, Family Desulfovibrionaceae [Estimate = -0.0418, 95% confidence interval [CI]: 0.9362-0.9826, P = 0.0007] exhibits stable and significant negative associations with ApoB levels. The inverse MR analysis did not find a significant causal effect of lipids on the intestinal flora. Additionally, no significant heterogeneity or horizontal pleiotropy for IVs was observed in the analysis. |
|  | c) | If relevant, consider translating estimates of relative risk into absolute risk for a meaningful time period | / | / |
|  | d) | Consider plots to visualize results (e.g. forest plot, scatterplot of associations between genetic variants and outcome versus between genetic variants and exposure) | 5 | Supplement Figure 1-2;5-6;9-10;13-14;17-18 |
| 12 | **Assessment of assumptions** |  |  |  |
|  | a) | Report the assessment of the validity of the assumptions | 5 | All included SNPs adhere to the criteria of independent inheritance (r2 < 0.01), no linkage disequilibrium (LD), and F>10 ("weak instruments" were excluded). For the inclusion of other exposed SNPs, see the Supplementary table S3 for details. |
|  | b) | Report any additional statistics (e.g., assessments of heterogeneity across genetic variants, such as *I^2^*, Q statistic or E-value) | 5 | We performed MR-Egger intercept tests to evaluate the possibility of horizontal pleiotropy, and no significant horizontal pleiotropy was observed. Similarly, MR-Egger showed that there was no horizontal pleiotropy between intestinal flora and lipids, which further proved the reliability of our causal inference results. Bias from horizontal pleiotropies could be largely ruled out by using Leave-one-out analysis, which shows that our MR results are stable and not driven by any single SNP (Supplementary figure 3,7,11,15,19). In Cochran's Q test for heterogeneity, both IVW and WM showed there was no heterogeneity among SNPs of intestinal flora. Funnel plot were used for visualization (Supplementary figure 4,8,12,16,20). |
| 13 | **Sensitivity analyses and additional analyses** |  |  |  |
|  | a) | Report any sensitivity analyses to assess the robustness of the main results to violations of the assumptions | 5 | We performed MR-Egger intercept tests to evaluate the possibility of horizontal pleiotropy, and no significant horizontal pleiotropy was observed. Similarly, MR-Egger showed that there was no horizontal pleiotropy between intestinal flora and lipids, which further proved the reliability of our causal inference results. Bias from horizontal pleiotropies could be largely ruled out by using Leave-one-out analysis, which shows that our MR results are stable and not driven by any single SNP (Supplementary figure 3,7,11,15,19). In Cochran's Q test for heterogeneity, both IVW and WM showed there was no heterogeneity among SNPs of intestinal flora. Funnel plot were used for visualization (Supplementary figure 4,8,12,16,20). |
|  | b) | Report results from other sensitivity analyses or additional analyses | / | / |
|  | c) | Report any assessment of direction of causal relationship (e.g., bidirectional MR) | 5 | To prevent reverse causality from interfering with the above results, we performed a reverse MR analysis with significant intestinal flora in two-sample MR studies as the outcome and lipids as the exposure on locus-wide significance level (Supplementary Table S7). There was no evidence for a causal effect of lipids on the intestinal flora. |
|  | d) | When relevant, report and compare with estimates from non-MR analyses | / | / |
|  | e) | Consider additional plots to visualize results (e.g., leave-one-out analyses) | 5 | Supplementary figure 3,7,11,15,19 |
|  | **DISCUSSION** |  |  |  |
| 14 | **Key results** | Summarize key results with reference to study objectives | 5,6 | The results indicated a potential causal association between 19 intestinal flora and dyslipidemia in humans. Genus Ruminococcaceae, Christensenellaceae, Parasutterella, Terrisporobacter, Parabacteroides, Class Erysipelotrichia, Family Erysipelotrichaceae, and order Erysipelotrichales were associated with higher lipids, whereas genus Oscillospira, Peptococcus, Ruminococcaceae UCG010, Ruminococcaceae UCG011, Dorea, and Family Desulfovibrionaceae were associated with lower lipids. Among them, Family Desulfovibrionaceae [Estimate = -0.0418, 95% confidence interval [CI]: 0.9362-0.9826, P = 0.0007] exhibits stable and significant negative associations with ApoB levels. The inverse MR analysis did not find a significant causal effect of lipids on the intestinal flora. Additionally, no significant heterogeneity or horizontal pleiotropy for IVs was observed in the analysis |
| 15 | **Limitations** | Discuss limitations of the study, taking into account the validity of the IV assumptions, other sources of potential bias, and imprecision. Discuss both direction and magnitude of any potential bias and any efforts to address them | 7 | We also need to acknowledge certain limitations in our study. Firstly, this study mainly included individuals of European ancestry, and additional validation is required when extending the results to other populations. Secondly, exposure factors such as diet and environment also have an impact on the composition and abundance of intestinal flora, we will treat it as the focus of our upcoming study. Lastly, despite the theoretical causal impact of certain bacterial groups, the specific mechanisms remain unclear. To elucidate the role of intestinal flora and its contribution to lipid homeostasis, both single flora transplantation and a substantial number of animal experiments are warranted. Our research team is currently engaged in related investigations to identify potential strategic targets for lipid level control. |
| 16 | **Interpretation** |  |  |  |
|  | a) | Meaning: Give a cautious overall interpretation of results in the context of their limitations and in comparison with other studies | 6,7 | The results indicated a potential causal association between 19 intestinal flora and dyslipidemia in humans. Genus Ruminococcaceae, Christensenellaceae, Parasutterella, Terrisporobacter, Parabacteroides, Class Erysipelotrichia, Family Erysipelotrichaceae, and order Erysipelotrichales were associated with higher lipids, whereas genus Oscillospira, Peptococcus, Ruminococcaceae UCG010, Ruminococcaceae UCG011, Dorea, and Family Desulfovibrionaceae were associated with lower lipids. Among them, Family Desulfovibrionaceae [Estimate = -0.0418, 95% confidence interval [CI]: 0.9362-0.9826, P = 0.0007] exhibits stable and significant negative associations with ApoB levels. The inverse MR analysis did not find a significant causal effect of lipids on the intestinal flora. Additionally, no significant heterogeneity or horizontal pleiotropy for IVs was observed in the analysis  The study suggested a causal relationship between intestinal flora and dyslipidemia. These findings will provide a meaningful reference to discover dyslipidemia for intervention to address the problems in the clinic. |
|  | b) | Mechanism: Discuss underlying biological mechanisms that could drive a potential causal relationship between the investigated exposure and the outcome, and whether the gene-environment equivalence assumption is reasonable. Use causal language carefully, clarifying that IV estimates may provide causal effects only under certain assumptions | 6,7 | we employed the large, publicly available GWAS database and applied MR analysis to explore the causal relationship between intestinal flora and lipids. In the present study, we identified a total of 19 lipid-related intestinal flora. Among them, a significant negative causal relationship exists between Desulfovibrionaceae and ApoB. Besides, no reverse causality was found by the reverse MR analysis.  Desulfovibrionaceae is an important anaerobic bacterium in the digestive tract. It can bind to human colonic mucin and high levels in mucosal samples of the large intestine. Researchers have noted a negative correlation between Desulfovibrio and obesity indicators such as BMI and waist[. An important characteristic of Desulfovibrio is its ability to perform dissimilatory sulfate reduction by utilizing sulfate as an electron acceptor for respiration, thereby producing hydrogen sulfide (H2S). As an important gas transmitter, H2S is involved in numerous biological processes, including posttranslational modifications of proteins by S-sulfhydration in the cardiovascular system and lipid metabolism. Some studies indicate that the reduction of H2S is associated with an accelerated occurrence of atherosclerosis. After feeding Cystathionine γ-lyase-deficient mice to a high-fat diet for 12 weeks, Mani observed significant disturbances in lipid metabolism and early atheromatous changes in the aorta. Treatment of these animals with the rapid H2S donor sodium hydrosulfide reduced the development of atherosclerosis.  This may suggest that future interventions on H2S could potentially serve as a viable direction for maintaining lipid metabolism homeostasis and slowing the development of atherosclerosis. However, at the current stage, how to manipulate H2S levels in a physiologically appropriate manner is a major concern. Desulfovibrionaceae as an important endogenous source of H2S, or targeting of Desulfovibrionaceae will help future studies in this regard.  Interestingly, metagenomics revealed that Desulfovibrio can produce acetic acid, which, as an important member of short-chain fatty acids (SCFAS), is undoubtedly essential for lipid metabolism homeostasis . Acetic acid can activate the AMP-activated protein kinase signaling pathway to regulate hepatic lipid metabolism. Moreover, the polymorphism of gut microbial communities, particularly those associated with lipid metabolic homeostasis, such as Coprococcus, Ruminococcus, Akkermansia, Roseburia, and Faecalibacterium, closely correlates with the relative abundance of Desulfovibrionaceae. The protective effects of Coprococcus , Ruminococcus, Akkermansia, Roseburia, and Faecalibacteriumare associated with the production of SCFAS. This phenomenon could have a synergistic effect with acetic acid produced by Desulfovibrionaceae, contributing to the maintenance of lipid metabolism homeostasis and the protection of host health. It is imperative to acknowledge that, while these mechanisms provide initial insights into the association between Desulfovibrionaceae and blood lipids, further investigation is still needed for a comprehensive understanding of the specific underlying mechanisms.  Additionally, ApoB functions as the primary transporter of LDL-C, and these two components are intricately connected within the organism. Elevated levels of LDL-C unquestionably expedite the progression of atherosclerosis, and our study indicates that certain intestinal flora may synergistically affect both. Oscillospira, an intestinal anaerobe, can utilize host glycans and produce butyrate. Butyrate plays a crucial role in maintaining metabolic homeostasis. In animal models of metabolic diseases, supplementation with butyrate reportedly confers numerous benefits, including reduced serum triglycerides, total cholesterol and glucose, and reduced weight gain in response to a high fat diet (HFD) This protective effect may be attributed to epigenetic effects through inhibition of histone deacetylases (HDACs). HDACs are a group of epigenetic modifying enzymes that remove acetyl groups from histone tails, thereby modifying chromatin structure and the accessibility of genes for transcription. HDACs regulate a variety of metabolic pathways and deregulation of HDACs has been associated with CVD. Apart from this. Butyrate can bind and activate the G protein-coupled (GPR) free fatty acid receptors (FFAR), influencing the release of gut hormones. These gut hormones may play an important role in appetite suppression and lipid metabolism.In our study, Parasutterella also could affect both Apo B and LDL-C. In a study on obesity, researchers found that Parasutterella could impact human fatty acid synthesis. This may exert a direct impact on ApoB production and LDL-C metabolism. Parasutterella colonies were also found to be significantly enriched in mice susceptible to obesity. Future interventions targeting Parasutterella may be a feasible way to combat obesity and maintain lipid homeostasis. Apart from this, our analysis complements the findings of Lee. Lee et al. found that Terrisporobacter could affect TG and HDL-C, We will further delineate the causal relationship between Terrisporobacter and ApoB and LDL-C. We are confident that our study can establish a more solid research foundation for future investigations.  In addition to the "bad cholesterol" mentioned above, HDL-C is widely recognized as the "good cholesterol" in our circulation. The latest research indicates that with each unit increase in HDL-C level, there is a corresponding 2–3% reduction in the risk of CVD. In the present MR analysis, we find a positive causal relationship between some intestinal flora and HDL-C, such as Erysipelotrichia. Erysipelotrichia is an important bacterium for maintaining intestinal health. Erysipelotrichia microflora transplantation has demonstrated great potential advantages in promoting intestinal regeneration after radiation. This important role in maintaining healthy gut homeostasis may emerge as a viable therapeutic option for atherosclerosis treatment in the future. Our results also suggest that Ruminococcaceae affects lipid metabolism. Priscilla et al. had observed a significant increase in the abundance of Ruminococcaceae in the control group compared to patients with atherosclerotic dyslipidemia . According to our analysis, this increase in abundance may regulate apolipoprotein and cholesterol, consequently exerting a protective effect on the host. To our surprise. We find for the first time a potential link between Dorea and TG. Dorea is a member of the family Lachnospiraceae which is reported to be strongly associated with lower TG levels in European and Chinese populations. Our study suggests that we cannot exclude the influence of Dorea on TG in this context, and we believe that our results can provide new evidence and confidence for the increasing of intestinal Dorea number in patients with dyslipidemia in the future. |
|  | c) | Clinical relevance: Discuss whether the results have clinical or public policy relevance, and to what extent they inform effect sizes of possible interventions | 5,6 | The results of this analysis provide support for the causal relationship between intestinal flora and serum lipids. These findings will provide a meaningful reference to discover dyslipidemia for intervention to address the problems in the clinic. |
| 17 | **Generalizability** | Discuss the generalizability of the study results (a) to other populations, (b) across other exposure periods/timings, and (c) across other levels of exposure | 7 | Our study also has some limitations. The GWAS data derived from individuals of European ancestry, with no representation of other ethnic populations. Hence, caution must be exercised when extrapolating our MR analysis outcomes to other groups as it may only be generalizable to European ancestry populations. |
|  | **OTHER INFORMATION** |  |  |  |
| 18 | **Funding** | Describe sources of funding and the role of funders in the present study and, if applicable, sources of funding for the databases and original study or studies on which the present study is based | 7 | Natural Science Foundation of Shandong Province (ZR2022QH103). |
| 19 | **Data and data sharing** | Provide the data used to perform all analyses or report where and how the data can be accessed, and reference these sources in the article. Provide the statistical code needed to reproduce the results in the article, or report whether the code is publicly accessible and if so, where | 7 | Only publicly available GWAS summary data were used in this work. All raw data for this study are publicly available in the IEU Open GWAS Project repository (([IEU OpenGWAS project (mercies. ac. UK)](https://gwas.mrcieu.ac.uk/)). Exposure dataset from MiBioGen  consortium (<https://mibiogen.gcc.rug.nl/>). 211 GM taxa (including nine phyla, 16 classes, 20 orders, 35 families, and 131 genera). Outcome dataset can be found here: https://mibiogen.gcc.rug.nl/ UK Biobank.  . |
| 20 | **Conflicts of Interest** | All authors should declare all potential conflicts of interest | 7 | We have no competing interests. |

This checklist is copyrighted by the Equator Network under the Creative Commons Attribution 3.0 Unported (CC BY 3.0) license.

1. Skrivankova VW, Richmond RC, Woolf BAR, Yarmolinsky J, Davies NM, Swanson SA, et al. Strengthening the Reporting of Observational Studies in Epidemiology using Mendelian Randomization (STROBE-MR) Statement. JAMA. 2021;under review.

2. Skrivankova VW, Richmond RC, Woolf BAR, Davies NM, Swanson SA, VanderWeele TJ, et al. Strengthening the Reporting of Observational Studies in Epidemiology using Mendelian Randomisation (STROBE-MR): Explanation and Elaboration. BMJ. 2021;375:n2233.
